# Radiolabelling an ^18^F biologic *via* facile IEDDA “click” chemistry on the GE FASTLab™ platform†

**DOI:** 10.1039/d1re00117e

**Published:** 2021-04-15

**Authors:** Louis Allott, Ala Amgheib, Chris Barnes, Marta Braga, Diana Brickute, Ning Wang, Ruisi Fu, Sadaf Ghaem-Maghami, Eric O. Aboagye

**Affiliations:** Comprehensive Cancer Imaging Centre, Faculty of Medicine, Department of Surgery and Cancer, Imperial College London, Hammersmith Hospital Du Cane Road London W12 0NN UK eric.aboagye@imperial.ac.uk; Positron Emission Tomography Research Centre, Faculty of Health Sciences, University of Hull Cottingham Road Kingston upon Hull HU6 7RX UK; Department of Biomedical Sciences, Faculty of Health Sciences, University of Hull Cottingham Road Kingston upon Hull HU6 7RX UK; Faculty of Medicine, Department of Surgery and Cancer, Imperial College London, Hammersmith Hospital Du Cane Road London W12 0NN UK

## Abstract

The use of biologics in positron emission tomography (PET) imaging is an important area of radiopharmaceutical development and new automated methods are required to facilitate their production. We report an automated radiosynthesis method to produce a radiolabelled biologic *via* facile inverse electron demand Diels–Alder (IEDDA) “click” chemistry on a single GE FASTLab™ cassette. We exemplified the method by producing a fluorine-18 radiolabelled interleukin-2 (IL2) radioconjugate from a *trans*-cyclooctene (TCO) modified IL2 precursor. The radioconjugate was produced using a fully automated radiosynthesis on a single FASTLab™ cassette in a decay-corrected radiochemical yield (RCY, d.c.) of 19.8 ± 2.6% in 110 min (from start of synthesis); the molar activity was 132.3 ± 14.6 GBq μmol^−1^. The *in vitro* uptake of [^18^F]TTCO-IL2 correlated with the differential receptor expression (CD25, CD122, CD132) in PC3, NK-92 and activated human PBMCs. The automated method may be adapted for the radiosynthesis of any TCO-modified protein *via* IEDDA chemistry.

## Abstract 2

A fully automated and efficient radiosynthesis of a novel interleukin-2 radioconjugate from a single FASTLab™ cassette.

## Graphical abstract

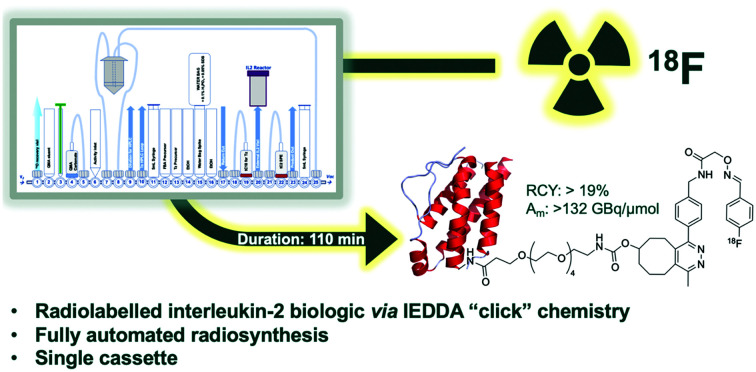

## Introduction

Positron emission tomography (PET) is a functional molecular imaging modality used in the clinic to inform the diagnosis and progression of diseases like cancer.^1,2^ Targeted radiopharmaceuticals are required to trace or quantify biological processes associated with disease states (*i.e.* energy metabolism, proliferation, receptor expression) and an active research and development community is working towards producing such molecules for clinical translation. The transition of radiopharmaceuticals from the laboratory bench into clinical evaluation requires automated radiosynthesis under good manufacturing processes (GMP) conditions, to produce consistent sterile patient doses and reduce radiation exposure to production staff.^3,4^ As we ask evermore complex clinical questions, the development of new radiopharmaceuticals with intricate and sensitive chemical structures often provide answers; however, their efficient radiolabelling and automated production can be challenging. The implementation of radiolabelled peptides and proteins as targeting vectors in nuclear medicine is increasing owed to their unparalelled targeted affinity, selectivity and specificity;^5^ however, few GMP compatible automated radiolabelling processes have been reported.^6,7^ We and others have developed various automated radiolabelling procedures for their production, including an automated procedure for copper catalysed azide alkyne cycloaddition (CuAAC) “click” chemistry for an ^18^F somatostatin analogue ([^18^F]FET-βAG-TOCA) which has progressed into phase III clinical trials;^8,9^ a generic automated radiolabelling procedure for the aluminium-[^18^F]fluoride method to produce [^18^F]NOTA-octreotide and [^18^F]NOTA-RGDfK radioconjugates and an automated procedure for solid-supported reductive amination radiochemistry.^10–12^

**Fig. 1:**
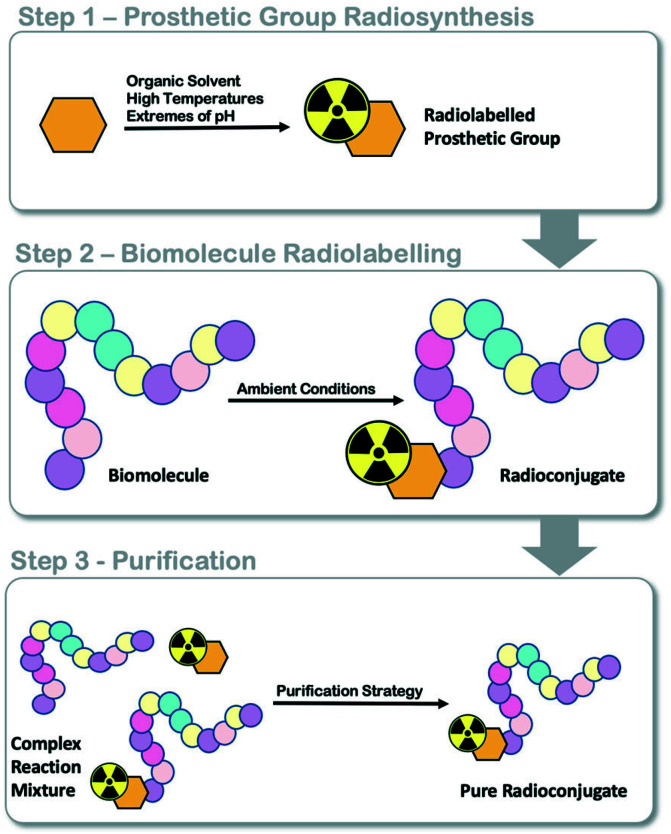
Schematic representation of fluorine-18 prosthetic group (PG) strategies for radiolabelling peptides and proteins.

Fluorine-18 prosthetic group (PG) strategies, where a small organic molecule is radiolabelled independently of a biomolecule and later conjugated under mild conditions, can be used to maintain the structural integrity of the protein (Fig. 1).^13^ Reported PG strategies and their automated procedures support the production of radioconjugates from micromolar (μmol) quantities of biomolecule precursors, typically peptides (*ca.* 1–4 kDa) which are inexpensive to synthesise in multi-milligram quantities.^8,14^ Targeted proteins (>5 kDa) are often made in small batches (μg to mg) as they are expensive to produce and are therefore radiolabelled at low total protein quantities (μg, nmol) to reduce cost, but also because of the challenge in separating the radioconjugate from the unlabelled biomolecule, which can negatively influence molar activity (*A*_m_). There are few examples where targeted proteins are radiolabelled with an ^18^F-PG using fully automated procedures, and to the best of our knowledge, no examples where an entire radiosynthesis has been accomplished on a single cassette-based automated platform.

The biologic interleukin-2 (IL2) is of great interest as it targets the IL2-receptor (IL2R) expressed on activated T-cells. IL2R is comprised of IL2Rα (CD25), IL2Rβ (CD122) and IL2Rγ (CD132) subunits resulting in a variable affinity receptor.^15^ Recombinant IL2 is commercially available as Proleukin™ (Novartis) and is used in the treatment of metastatic renal cell carcinoma and melanoma; typically administered as intravenous infusion or subcutaneous injection.^16,17^ Targeting the high affinity CD25 using IL2-based radioconjugates could potentially enable the detection of T-cell activation in solid tumours and, thus monitoring of response to immunotherapy.^3,18,19^ Gialleonardo *et al.* (2012) reported a fluorine-18 IL2 radioconjugate ([^18^F]FB-IL2) which was synthesised using the *N*-succinimidyl-4-[^18^F]fluorobenzoate ([^18^F]SFB) prosthetic group and later translated the radiochemistry into a GMP compatible production to support a clinical trial.^6,20^ Unfortunately, the lengthy radiosynthesis of [^18^F]SFB and the complexity of radiolabelling proteins using automated platforms required the use of two hot-cells and two automated radiosynthesis platforms. This process is not attractive to GMP production facilities. DeVries *et al.* are addressing this concern and have developed metal-based radiolabelling strategies;^21^ but the question remains, if we can develop an ^18^F-PG strategy for the automated production of proteinous radioconjugates using a single cassette-based platform?

The use of inverse electron demand Diels–Alder (IEDDA) chemistry to radiolabel a protein modified with a *trans*-cyclooctene (TCO) moiety by ligating an ^18^F-tetrazine prosthetic group has been reported but an automated radiolabelling procedure has not been described.^22^ We report a fully automated ^18^F-PG strategy used to radiolabel TCO-modified IL2 using IEDDA “click” chemistry. We believe this to be the first fully automated radiosynthesis of an IL2 radioconjugate using an ^18^F-PG strategy on a single cassette-based automated platform. The reported method may be adapted for use in radiolabelling any TCO-modified protein and “noteworthy considerations” are suggested throughout for translating this work to other biomolecules of interest.

## Experimental procedures

Detailed materials and methods appear in the ESI.† Radiochemical yields (RCY) are decay corrected (d.c.) to the start of radiosynthesis, in line with the “Consensus nomenclature rules for radiopharmaceutical chemistry – Setting the record straight”.^23^ Experimental procedures for the synthesis of compounds **1–5** and their characterisation by NMR and mass spectrometry appear in the ESI† (Fig. S1–S12). Experimental procedure for *in vitro* metabolite analysis, cell culture and flow cytometry are reported in the ESI† (Fig. S22–S24).

## Materials and methods

Anhydrous solvents and reagents were purchased from Sigma Aldrich (Gillingham, UK) and were used without additional purification. TCO-PEG_4_-NHS ester was purchased from Jena Bioscience (Jena, Germany). Proleukin™ was procured from Novartis Pharmaceuticals (London, UK). Flash column chromatography purification was performed on silica gel (Merck Kieselgel 60 F254 320–400 mesh). [^18^F]Fluoride was produced by a GE PETtrace cyclotron by 16 MeV irradiation of enriched [^18^O]H_2_O target, supplied by Alliance Medical Radiopharmacy Ltd (Warwick, UK). Automated radiosynthesis were performed using the GE FASTlab™ automated synthesis module (GE Healthcare Life Sciences, Amersham, UK). Solid phase extraction (SPE) cartridges were purchased from Waters (Elstree, Hertfordshire, UK) and used according to the manufacturers recommended guidelines. Semi-preparative RP-HPLC was performed using a Shimadzu LC20-AT pump attached to a custom-built system, equipped with an Agilent Eclipse XDB-C18, 5 μ (250 × 9.4 mm) column using an isocratic mobile phase of MeCN (44%), H_2_O (56%) and 0.1% H_3_PO_4_ (14.8 M) at a flow rate of 3 mL min^−1^. Reaction efficiency and radioactive product identity was determined by RP-HPLC using an Agilent 1200 series instrument connected to a flow-ram detector (Lablogic, Sheffield, UK). This study used a PC3 cell line, gifted from Prof. Charlotte Bevan, Imperial College London and a NK-92 cell line purchased from ATCC (Teddington, UK). Peripheral blood mononuclear cells (PBMCs) were isolated from whole blood samples obtained from healthy volunteers under the approval of the West London Research Ethics Committee (Reference 10/H0707/7 and 12/WA/0196).

### Synthesis of TCO-PEG_4_-IL2

To each of three vials of Proleukin™ (18 × 106 IU, ∼1.3 mg) was added water (250 μL), which was combined into a single vial to give a Proleukin™ (3.96 mg, 255 nmol) in water (750 μL, 5.28 mg mL^−1^). A Zeba™ spin desalting column (7 kDa MWCO, 5 mL) was equilibrated with a pH 8 bioconjugation solution containing NaHCO_3_ (0.1 M) and SDS (0.05 % w/v), as per the manufacturers recommended equilibration protocol. The Proleukin™ solution was exchanged into the NaHCO_3_/SDS solution and protein recovery was determined to be quantitative by UV-vis (nanodrop). A fresh stock of TCO-PEG_4_-NHS ester was prepared in DMF (255 nmol μL^−1^) and an aliquot (10.6 μL) was added to the Proleukin™ solution in a 12 : 1 ratio (TCO-PEG_4_-NHS ester : Proleukin™). The reaction was gently shaken for 2 h at ambient temperature. A Zeba™ spin desalting column (7 kDa MWCO, 5 mL) was equilibrated in a storage solution containing SDS (0.05% w/v) and PSB. The Proleukin™ reaction mixture was loaded onto the column and purified. The resulting solution contained TCO-PEG_4_-IL2, and the protein content was determined by UV-vis spectroscopy (nanodrop) and BCA assay; aliquots of 200 μg were prepared for radiolabelling and stored at −20 °C. The number of TCO moieties per molecule of IL2 was determined by nanodrop and the full experimental procedure described in the ESI.†

### Automated radiosynthesis of [^18^F]FB-Tz

A previously reported radiosynthesis for automating reductive amination radiochemistry using solid-supported cyanoborohydride cartridges was adapted for the synthesis of [^18^F]FB-Tz.^10^ In summary, [^18^F]FBA was synthesised to which precursor 1 (35 mg) in MeCN (1.5 mL) and triethylamine (40 μL) was added. After warming and reduction using the BH_3_CN^−^ cartridge following the published protocol, the reaction mixture was diluted in phosphate buffer (5 mL, pH 2.4) and purified by semi-preparative HPLC. The cut peak was diluted in phosphate buffer (45 mL, pH 2.4) and trapped on a tC18 plus SPE cartridge which was subsequently flushed with nitrogen. [^18^F]FB-Tz was eluted with EtOH.

### Automated radiosynthesis of [^18^F]FBoxTz

A detailed description of the automated radiosynthesis setup can be found in the ESI† (Fig. S14). This radiosynthesis required the water bag (position 15) to be modified to include H_3_PO_4_ (0.1% v/v) and SDS (0.05% w/v). A GE FASTLab™ system was programmed to trap aqueous [^18^F]fluoride from [^18^O]water on a QMA bicarbonate cartridge (position 4–5) which was eluted into the reactor using a solution containing KHCO_3_ (3.5 mg mL^−1^, H_2_O 200 μL) and Kryptofix-222 (6.0 mg mL^−1^, MeCN 800 μL). The [^18^F]fluoride was dried at 120 °C (9 min) and 70 °C (5 min) before the addition of 4-formyl-*N*,*N*,*N*-trimethylanilinium triflate (3 mg in 1.4 mL MeCN). The reaction was heated to 90 °C for 6.6 min to synthesise [^18^F]FBA. After cooling, a solution containing 3 (8 mg in 1 mL MeCN) and aniline hydrochloride (6 mg in 400 μL H_2_O) was added to the [^18^F]FBA in the reactor and warmed to 40 °C for 10 min to synthesise [^18^F]FBoxTz. The reaction mixture was diluted for semi-preparative HPLC purification (7.5 mL H_2_O + 0.1% H_3_PO_4_). The isolated [^18^F]FBoxTz (*t*_R_ = 21–24 min) was cut into a vial containing a dilution mixture (35 mL H_2_O + 0.1% H_3_PO_4_) and returned to the FASTLab™ for trapping on a tC18 SPE. The trapped [^18^F]FBoxTz was washed with the water bag solution (2 × 5 mL) and dried under N_2_. [^18^F]FBoxTz was eluted from the SPE cartridge in ethanol. The sequence continued for the automated radiosynthesis of [^18^F]TTCO-IL2.

### Automated radiosynthesis of [^18^F]TTCO-IL2

[^18^F]FBoxTz was eluted into an off-board reactor with ethanol (500 μL) containing TCO-PEG_4_-IL2 (200 μg, 2–3 mg mL^−1^) and allowed to react for 15 min at ambient temperature. The reaction mixture containing [^18^F]TTCO-IL2 was diluted with the water bag solution (9 mL) and loaded onto a tC2 SPE cartridge. Unreacted [^18^F]FBoxTz was eluted to waste by washing with a 50% EtOH solution. The desired [^18^F]TTCO-IL2 was eluted from the cartridge using 100% EtOH. The minimum SA was determined by the following equation: [radioactivity in product vial (MBq)]/200 μg = specific activity, SA (MBq μg^−1^); or by integrating the area under the radioactive peak at the 210 nm wavelength and comparing area to a reference standard of TCO-PEG_4_-IL2. Radiochemical purity was determined by radio-HPLC (*t*_R_ = 08:48 mm:ss) and radio-TLC (*R*_f_ = 0).

### Radiochemical stability of [^18^F]TTCO-IL2

A vial of [^18^F]TTCO-IL2 in the standard formulation was synthesised using the automated procedure starting from a high activity of [^18^F]fluoride (19.6 ± 2.4 GBq, *n* = 3). An aliquot of radioactivity (4.4 ± 0.9 MBq) was diluted in 0.1% TFA (500 μL) and analysed by HPLC for each 0, 0.5, 1, 2, 3, 4, 5 and 6 h time point. Radio-TLC was also used (iTLC silica sheet, mobile phase: 2 : 1 EtOAc : hexane) for 1, 2, 3, 4, 5 and 6 h time points. The experiment was repeated three times from separate productions.

### *In vitro* radioactive uptake

To assess the *in vitro* uptake of [^18^F]TTCO-IL2, 72 h PHA-stimulated PBMCs (approximately 1 × 10^6^ cells per tube) were incubated with 0.74 MBq of [^18^F]TTCO-IL2 at 37 °C and 5% CO_2_. PC3 cells were seeded in a 6-well plate at a seeding density of 0.3 × 10^6^ cells per well 24 hours prior to administration of [^18^F]TTCO-IL2. Approximately 1 × 10^6^ of NK-92 cells were incubated with 0.74 MBq of [^18^F]TTCO-IL2 at room temperature. After 1 hour, cells were washed twice with 500 μL of ice-cold PBS and lysed with 1 mL ice-cold RIPA buffer (ThermoFisher) for 10 minutes on ice. Cell-bound radioactivity was measured, and decay corrected using Packard Cobra II gamma counter (Perkin Elmer). The radiopharmaceutical uptake was normalised to total cellular protein. Uptake was expressed as % radioactivity per mg protein. To determine the effect of [^18^F]TTCO-IL2 on IL2R signalling, NK-92 cells were incubated with 0.74 MBq [^18^F]TTCO-IL2 or unlabelled recombinant IL2 (214 ng mL^−1^) for 1 hour at 37 °C and 5% CO_2_. Cells were washed as previously described and fixed with 4% paraformaldehyde for 20 minutes before proceeding with phosphorylated-STAT5 (Tyr694) analysis using flow cytometry. Data were summarised and analysed using Prism GraphPad 7 software. Statistical analysis was performed using an unpaired two-tailed *t*-test.

## Results and discussion

The use and automation of ^18^F-PG strategies to radiolabel proteins is challenging for several reasons:

1. ^18^F-PG strategies can be challenging to synthesise. Depending on the PG, multistep radiosynthesis and purification are required to access the desired ^18^F-PG. The PG needs to be produced in high *A*_m_ for radiolabelling biomolecules through second order reactions to avoid self-competition at reactive sites.

2. ^18^F-PGs can be unstable. *N*-Hydroxysuccinimide esters (*e.g.* [^18^F]SFB) rapidly hydrolyse in aqueous media at the pH required for efficient amide bond formation with lysine amino acids (8–9 pH). Competitive hydrolysis reduces the efficiency in radiolabelling the molecule of interest, impacting both radiochemical yield (RCY) and potentially, molar activity (*A*_m_).

3. Cassette-based automated platforms have a limited capacity. These platforms are primarily designed for small-molecule radiochemistry where S_N_^2 18^F-fluorination is followed by deprotection and a purification step; radiolabelling biomolecules through ^18^F-PG strategies is more complex in terms of i) number of synthetic steps, ii) types of purification strategy, iii) low reagent concentrations and thus iv) low volume reagent handling.

“Click” chemistry has been proved to be an effective strategy for radiolabelling complex molecules.^8,9,14,22,24,25^ We proposed that the IEDDA “click” reaction could address each of these limitations and produce a fully automated single-cassette based method for radiolabelling biomolecules.^22^ Given the challenges faced in producing an efficient radiosynthesis of [^18^F]FB-IL2, and its clinical relevance, we developed an automated IEDDA “click” method to radiolabel a novel TCO-modified IL2 conjugate (TCO-PEG_4_-IL2) that we produced for this project.

### Developing a TCO-modified IL2 conjugate

The abundant availability of TCO reagents allowed for a simple route to accessing a TCO-containing IL2 precursor by conjugating commercially available TCO-PEG_4_-NHS ester to recombinant IL2 (Proleukin™). In brief, the lyophilised recombinant IL2 (1.3 mg vial) was reconstituted in water to a concentration of 5 mg mL^−1^ and exchanged into a sodium bicarbonate (0.1 M, pH 8) conjugation medium using Zeba™ spin desalting columns. The TCO-PEG_4_-NHS ester was added to the reaction mixture in a 12 : 1 molar ratio, similar to that used in the development of [^99m^Tc]Tc-HYNIC-IL2.^26^ After gentle shaking for 2 hours at room temperature, the bioconjugate was purified by Zeba™ spin desalting column. The protein concentration was determined by bicinchoninic acid (BCA) assay and typical yields were *ca.* 60%. The average number of TCO moieties per molecule of IL2 was 0.74 ± 0.02, determined by UV-vis spectroscopy. The TCO-PEG_4_-IL2 bioconjugate was aliquoted into 1.5 mL microcentrifuge tubes for radiolabelling and stored at −20 °C. HPLC analysis of TCO-PEG_4_-IL2 resulted in a single peak at 08:18 mm:ss (ESI† Fig. S19).

It was considered that functionalising IL2 with TCO-PEG_4_ could change the biological properties of the protein. Prodrug strategies for IL2 have been developed by decorating the surface of the protein with large PEG chains to modulated the affinity, target selectivity and *in vivo* pharmacokinetics of the drug.^27^ Additionally, small modifications to the protein structure of IL2 have been reported to influence binding modes.^28–30^ To investigate the influence of our modification to IL2, the *in vitro* biological properties of [^18^F]TTCO-IL2 have been evaluated and are reported later.

#### Noteworthy consideration

Site-specific modification of proteins can potentially overcome poor biological performance by producing discrete molecular structures where key binding sites remain intact.^31–33^

### Development of a convenient ^18^F-tetrazine prosthetic group

**Scheme 1:**
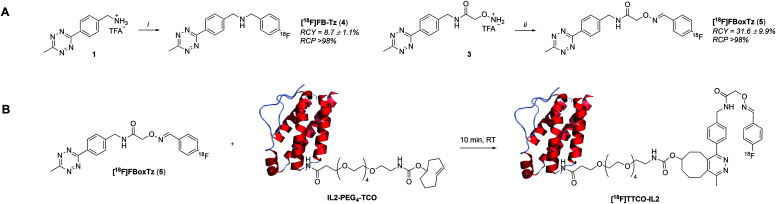
A) Two radiosynthetic routes to ^18^F-tetrazines: *N*-(4-[^18^F]fluorobenzyl)-1-(4-(6-methyl-1,2,4,5-tetrazin-3-yl)phenyl)methanamine ([^18^F]FB-Tz) and *E*-2-(((4-[^18^F]fluorobenzylidene)amino)oxy)-*N*-(4-(6-methyl-1,2,4,5-tetrazin-3-yl)benzyl)acetamide ([^18^F]FBoxTz). Reaction conditions: i) [^18^F]FBA, solid-supported cyanoborohydride cartridge, MeCN/H_2_O; ii) [^18^F]FBA, aniline hydrochloride, MeCN/H_2_O, 40 °C, 10 min. B) The reaction between [^18^F]FBoxTz and TCO-PEG_4_-IL2 to produce the radioconjugate [^18^F]TTCO-IL2.

There are many examples of ^18^F-radiolabelled tetrazines produced by different radiosynthesis methods and in variable RCY, fuelled by the increased interest in pre-targeted immuno-PET;^34–37^ however, new ^18^F-tetrazines were developed specifically as PGs that could be accessed in a high RCY, *A*_m_ and in few radiosynthetic steps. The direct ^18^F-fluorination of a tetrazine is notoriously difficult due to instability in the harsh reaction conditions required to promote nucleophilic chemistry. The modular assembly of two novel ^18^F-tetrazines was therefore favoured and reductive amination chemistry, as well as oxime chemistry, were evaluated (Scheme 1A). The radiosynthesis of *N*-(4-[^18^F]fluorobenzyl)-1-(4-(6-methyl-1,2,4,5-tetrazin-3-yl)phenyl)methanamine ([^18^F]FB-Tz) used simple reductive chemistry reaction between an amine-containing tetrazine (1) and 4-[^18^F]fluorobenzaldehyde ([^18^F]FBA). The automated radiochemistry was derived from our previously published work on the development solid-supported cyanoborohydride reducing agent cartridges.^10^ [^18^F]FB-Tz was successfully produced in a 8.7 ± 1.1% RCY (d.c.) using a fully automated procedure in 80 min. To compare the influence of reaction chemistry on the overall radiochemical properties of the PG, we developed *E*-2-(((4-[^18^F]fluorobenzylidene)amino)oxy)-*N*-(4-(6-methyl-1,2,4,5-tetrazin-3-yl)benzyl)acetamide ([^18^F]FBoxTz) to utilise oxime chemistry.

**Fig. 2:**
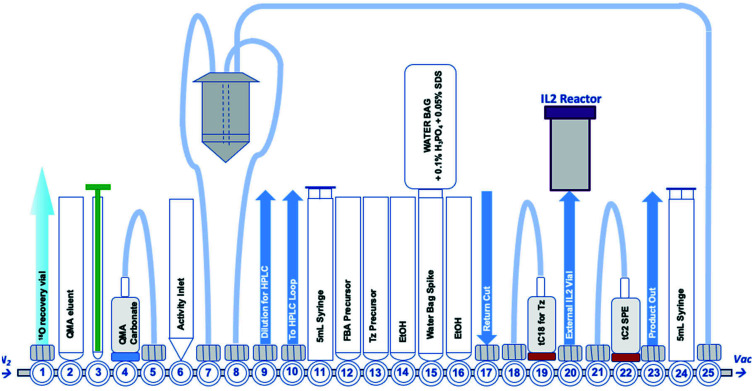
A schematic representation of the GE FASTLab™ cassette developed for the automated radiosynthesis of [^18^F]TTCO-IL2; a detailed description of the cassette setup appears in the ESI† (Fig. S14).

[^18^F]FBoxTz removed the need for an on-board reducing agent by exploiting oxime ligation between the aminoxy functional group and [^18^F]FBA. The radiochemistry was simple to automate and produced [^18^F]FBoxTz in a 31.6 ± 9.9% RCY (d.c.) in 85 min. In brief, [^18^F]fluoride was dried and [^18^F]FBA synthesised and precursor **3** added along with aniline hydrochloride as a catalyst. The resulting [^18^F]FBoxTz was purified by semi-preparative HPLC and the radioactive product reformulated using a tC18 solid phase extraction (SPE) cartridge, which was eluted with ethanol. The [^18^F]FBoxTz radiosynthesis not only gave a higher RCY (d.c.) compared to [^18^F]FB-Tz (*ca.* 32% *vs.* 9%), the purity profile was much improved and therefore fewer competing tetrazine impurities would be introduced into the IEDDA “click” step. The low RCY of [^18^F]FB-Tz was likely due to instability of the tetrazine under reducing conditions, converting into an unreactive dihydrotetrazine.^38^ Additionally, the radiosynthesis used fewer positions on the FASTLab™ cassette which would allow greater flexibility in implementing the entire radiolabelling method onto the single cassette (Fig. 2).

#### Noteworthy consideration

With advances in late-stage fluorination reactions, it will likely be possible to synthesise an ^18^F-tetrazine with appropriate characteristics (RCY, *A*_m_) in a single step.^39,40^ This would further reduce the overall complexity of the sequence and allow for even more flexibility in the cassette and sequence design. A semi-preparative HPLC system is not included as standard with the FASTLab™ platform and here, a custom-build HPLC module and complementary software was used to purify [^18^F]FBoxTz; the loading of crude reaction mixture was performed by syringe using the FASTLab™ cassette and sequence. The feasibility of this will depend upon the HPLC module available, but should be compatible if the system contains a Luer-lock fitted injection loop (≥10 mL).

### Automating the IEDDA “click” reaction

With an efficient ^18^F-tetrazine radiosynthesis developed, the cassette and sequence were expanded to automate the IEDDA “click” chemistry (Fig. 2). This reaction occurs quickly at ambient temperature, at low concentration of reagents and in the absence of metal catalysts meaning there were few variables to optimise other than the quantity of TCO-PEG_4_-IL2 precursor. A key radiochemical parameter for IL2 radioconjugates is a high final *A*_m_; IL2 is an agonist and there are reports of effective low-dose IL2 therapy (100 μg per dose).^41,42^ There is a precedent for PET and SPECT IL2 radioconjugates to been administered at <50 μg of total protein per dose, therefore we aimed to develop a similar protocol for this IL2 radioconjugate.^3,18,19,43^ The automated procedure and cassette layout for [^18^F]FBoxTz was adapted to elute the ^18^F-PG in ethanol into an external Wheaton vial containing the TCO-PEG_4_-IL2 precursor which reacted for 15 min before purification; purification of the radioconjugate is described later in this manuscript. The efficiency of the IEDDA “click” reaction was monitored by HPLC and radio-TLC. The full radiosynthesis is described in the ESI† (Fig. S14).

#### Noteworthy consideration

IL2 is a lipophilic protein and is stable in 100% ethanol therefore, no special attention was paid to the organic solvent content of the IEDDA “click” reaction. Depending on the biomolecule in question, this may be critical to its stability. It is possible to reduce the elution volume by using smaller SPE cartridges and more carefully considered elution volumes.

A maximum quantity of TCO-PEG_4_-IL2, 200 μg per radiolabelling reaction was investigated to produce clinically relevant *A*_m_ of [^18^F]TTCO-IL2. The automated synthesis produced [^18^F]TTCO-IL2 in 19.8 ± 2.6% RCY (d.c.) (*n* = 14) within 110 min from the start of synthesis in >98% RCP. The radiochemical yield was not influenced by the starting activity of [^18^F]fluoride, however the molar activity increased linearly (*R*^2^ = 0.9744) with starting activities ranging from 1.0–21.8 GBq (ESI,† Fig. S15). Three productions using 19.6 ± 2.4 GBq of [^18^F]fluoride gave an *A*_m_ of 132.3 ± 14.6 GBq μmol^−1^ (SA: 8.5 ± 0.8 MBq μg^−1^) which is sufficient for biological evaluation and clinical studies; in theory, a patient dose could be administered 2.5 h post radiosynthesis and still be within the 50 μg per dose IL2 limit. The recovery of the radioconjugate from the cassette was high, with negligible radiolabelled protein binding to plastic consumables.

#### Noteworthy consideration

Conditioning plastics for low protein binding with human serum albumin (HSA) or bovine serum albumin (BSA) is a simple solution to implement to aid recovery of a radioconjugate.

It was assumed that reducing the quantity of TCO-PEG_4_-IL2 precursor from 200 μg to 100 μg would improve the *A*_m_ if the IEDDA “click” efficiency remained the same. This hypothesis was tested by reducing the precursor quantity and reaction concentration by half; *A*_m_ did not improve as the overall RCY was diminished by 50% (Table 1). A TCO-PEG_4_-IL2 precursor quantity of 200 μg was maintained for all further experiments.

**Table 1:** Key radiochemical parameters (SA, *A*_m_ and RCY) for the radiosynthesis of [^18^F]TTCO-IL2, including the influence of TCO-PEG_4_-IL2 precursor quantity. Data presented as mean ± SD and experiments were performed in

	Entry 1	Entry 2	Entry 3
TCO-PEG_4_-IL2 (μg)	100^a^	200^b^	200^a^
Starting activity of [^18^F]fluoride (GBq)	7.5 ± 1.5	6.9 ± 2.2	19.6 ± 2.4
Specific activity (MBq μg^−1^)	3.8 ± 1.5	3.5 ± 1.2	8.5 ± 0.8
Molar activity (GBq μol^−1^)	59.4 ± 22.7	54.3 ± 18.0	132.3 ± 14.6
Radiochemical yield (% RCY, d.c.)	5.1 ± 1.6	10.2 ± 1.2	8.7 ± 0.7

a *n* = 3.

b *n* = 11 repeats.

### Purification of [^18^F]TTCO-IL2

Selecting an appropriate purification strategy for a radioconjugate is specific to the physicochemical properties of the protein of interest. IL2 radioconjugates have been effectively purified by tC2 SPE, a strategy made possible by the stability of the biomolecule to high concentrations of organic solvents.^6,44^ This is not the case for all proteins, which can denature under such conditions; however, some small protein fragments (*e.g.* affibody molecules) and DNA aptamers can tolerate high concentrations of organic solvent.^22,45^ [^18^F]TTCO-IL2 was purified by tC2 by adapting previously described procedures.^6,46,47^ This method removed unreacted [^18^F]FBoxTz and small-molecule impurities independently of [^18^F]TTCO-IL2, which was finally eluted into a formulation vial in >98% radiochemical purity (RCP).

#### Noteworthy consideration

While an SPE strategy was adapted for our application, there is sufficient space on the FASTLab™ cassette to incorporate a second semi-preparative HPLC purification using a biocompatible mobile phase. Alternatively, the automation of a size-exclusion purification method using commercially available Luer-lock syringe driven cartridges may provide an elegant alternative.

### Summary of complete automated radiosynthesis

**Fig. 3:**
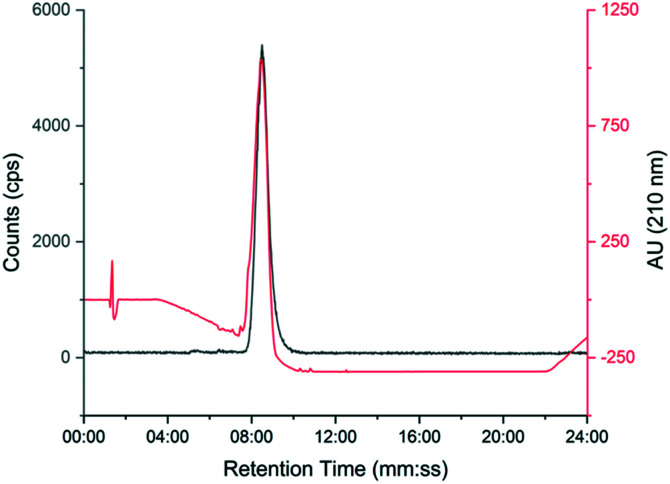
Representative HPLC chromatogram showing [^18^F]TTCO-IL2 (grey line) co-injected with non-reactive TCO-PEG_4_-IL2 (red line).

The automated radiosynthesis was simple to set up using commercially available components and reliably produced large batches of [^18^F]TTCO-IL2 (1.7 ± 0.2 GBq, *n* = 3). The radiosynthesis from [^18^F]fluoride to formulated [^18^F]TTCO-IL2 was complete in 110 min and the product was radiochemically stable (99.9 ± 0.1%) for the duration of testing (6 h) (ESI† Fig. S20 and S21). The identity of [^18^F]TTCO-IL2 was confirmed by radio-HPLC and a representative chromatogram is shown in Fig. 3. The RCY (d.c.) was 19.8 ± 2.6% (*n* = 14) and the molar activity was 132.3 ± 14.6 GBq μmol^−1^ from 19.6 ± 2.4 GBq of [^18^F]fluoride. Since completing this study, we have successfully produced GMP grade precursor **3** through a commercial research organisation to support future clinical translation of this strategy.

### *In vitro* evaluation of [^18^F]TTCO-IL2

[^18^F]TTCO-IL2 was evaluated *in vitro* against IL2R positive and negative cell lines, to confirm the retention of biological activity after radiolabelling. This was important to assess if any component of the conjugation or radiolabelling procedure had negatively influenced the integrity of IL2; additionally, this is the first time a TCO modified IL2 conjugate has been described and it was important to determine if TCO-PEG_4_ had perturbed its receptor recognition.

**Fig. 4:**
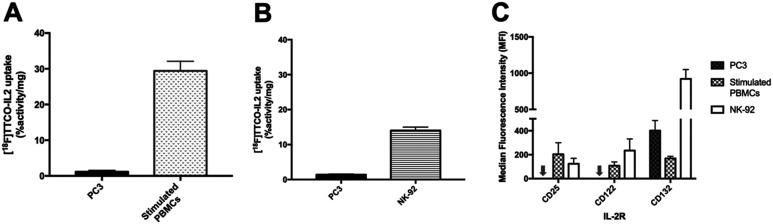
A) *In vitro* uptake of [^18^F]TTCO-IL2 (0.74 MBq, 85 ng–3.2 μg protein) in PC3 and PHA-stimulated PBMCs (72 h) at 37 °C. Two independent experiments were performed for PBMCs and PC3 (*n* = 6). B) *In vitro* uptake of [^18^F]TTCO-IL2 (0.74 MBq, 85–264 ng protein) in PC3 and NK-92 at room temperature (RT). Three independent experiments were performed from PC3 and NK-92 (*n* = 6). Data expressed as mean ± SEM. Significant difference between PC3 and NK-92/PHA-stimulated PBMCs is indicated by **** (*p* < 0.0001). Statistical difference is analysed using unpaired *t*-test. C) Surface expression of IL2R (CD25, CD122, CD132) in PC3 cells, NK-92 cells treated with 6.1 ng mL^−1^ (100 U mL^−1^) IL-2, and PHA-stimulated PBMCs (72 h) – arrow represents PC3 cell line with 0 MFI. Data are presented as median fluorescence intensity (MFI) from five donors for PBMCs and three independent experiments for PC3 and NK-92 cells.

The *in vitro* uptake of [^18^F]TTCO-IL2 in cell lines with differential IL2R expression was investigated (Fig. 4A and B). NK-92 cells and human PBMCs were stimulated to upregulate CD25 and consequently form the high affinity IL2R. PC3 prostate cancer cell line did not express CD25 and CD122, and therefore was used as a negative control. IL2R expression was confirmed by FACS analysis (Fig. 4C). The *in vitro* uptake of [^18^F]TTCO-IL2 is in agreement with the differential expression of CD25 in stimulated PBMCs, NK-92 and PC3 cells, and comparable to other IL2-based PET probes.^21^ Uptake was low in CD25 and CD122 negative PC3 cells indicating negligible non-specific binding. Analysis of phosphorylated STAT5 (Tyr694), critical for IL2 signalling, in NK-92 cells incubated with a dose of [^18^F]TTCO-IL2 (which also contains TCO-PEG_4_-IL2) demonstrated insignificant effect on STAT5 phosphorylation compared to recombinant IL2 at the same concentration (Fig. S26†). This suggests that [^18^F]TTCO-IL2 may not elicit an immune response when administered at a low concentration. The metabolic profile of [^18^F]TTCO-IL2 was determined *in vitro* using human liver microsomes (HLM) and mouse liver S9. The radioconjugate was stable (*ca.* 92% parent remaining in 60 min) for both species, although extraction efficiencies were low (19.5 ± 1.5% for HLM and 27.5 ± 3.3% for mouse liver S9), likely resulting from precipitation or aggregation of [^18^F]TTCO-IL2 caused by the extraction process from the biological milieu (Fig. S22†).

## Conclusion

To exemplify the utility of adapting IEDDA “click” chemistry to radiolabel proteins in a fully automated, single-cassette based procedure, we describe a method for radiolabelling a TCO-modified IL2 protein using the GE FASTLab™ platform. [^18^F]TTCO-IL2, a biologically active radioconjugate towards the IL2R was produced in 110 min from a single-cassette radiosynthesis. The *in vivo* evaluation of [^18^F]TTCO-IL2 is underway, but early data suggests uptake in target enriched tissues such as the lymphatic system; given the complexity of biological models of immune response, these data will be reported elsewhere. It is hoped that that the work presented here will be useful to the PET radiochemistry community for automating the radiolabelling of biomolecules of interest.

## Conflicts of interest

There are no conflicts to declare.

## Supplementary Material

RE-006-D1RE00117E-s001
