# Supplementary material for: Radiolabelling an 18F biologic via facile IEDDA “click” chemistry on the GE FASTLab™ platform
Source: React Chem Eng. 2021 Apr 15;6(6):1070–8. doi: 10.1039/d1re00117e (PMC8167423; doi:10.1039/d1re00117e)
Supplement: RE-006-D1RE00117E-s001 [file RE-006-D1RE00117E-s001.pdf]

Electronic Supplementary Material (ESI) for Reaction Chemistry & Engineering.
This journal is © The Royal Society of Chemistry 2021

RSC Reaction Chemistry and Engineering

Radiolabelling an ^{18}F biologic *via* facile IEDDA "click" chemistry on the GE FASTLab™ platform

Louis Allott[1,3,4‡], Ala Amgheib[1,2‡], Chris Barnes[1], Marta Braga[1], Diana Brickute[1], Ning Wang[1], Ruisi Fu[1], Sadaf Ghaem-Maghami[1,2] and Eric O. Aboagye[1*]

1. Comprehensive Cancer Imaging Centre, Faculty of Medicine, Department of Surgery and Cancer, Imperial College London, Hammersmith Hospital, Du Cane Road, London, W12 0NN
2. Faculty of Medicine, Department of Surgery and Cancer, Imperial College London, Hammersmith Hospital, Du Cane Road, London, W12 0NN
3. Positron Emission Tomography Research Centre, Faculty of Health Sciences, University of Hull, Cottingham Road, Kingston upon Hull, HU6 7RX
4. Department of Biomedical Sciences, Faculty of Health Sciences, University of Hull, Cottingham Road, Kingston upon Hull, HU6 7RX

‡ Equal contribution from authors

Supporting Information

Contents

1.0 Materials and methods……………………………………………………..……Page 02
2.0 Synthesis and characterisation...……………………………………………………Page 03
3.0 Determining the number of TCO moieties per molecule of IL2…………………...Page 12
4.0 Radiosynthesis………………………………………………………………...…Page 14
5.0 HPLC and TLC Chromatograms……………………………………………….....Page 17
6.0 Radiostability assays…. ………………………………………………………..…Page 19
7.0 *In vitro* Metabolite analysis……………...…………………………………………Page 20
8.0 Cell culture………………………………………………………………………....Page 20
9.0 Flow cytometry……………………..………………………………………………Page 21

1.0 Materials and Methods

Anhydrous solvents and reagents were purchased from Sigma Aldrich (Gillingham, UK) and were used without additional purification. TCO-PEG_4-NHS ester was purchased from Jena Bioscience (Jena, Germany). Proleukin™ was precured from Novartis Pharmaceuticals (London, UK). Flash column chromatography purification was performed on silica gel (Merck Kieselgel 60 F254 320–400 mesh). Thin Layer Chromatography (TLC) was performed on Merck aluminium-backed plates pre-coated with silica (0.2mm, 60 F254) which were visualised by quenching of ultraviolet fluorescence (λ = 254 and 365 nm). ^{1}H, ^{13}C and ^{19}F NMR spectra were obtained using a Bruker 400 MHz spectrometer operating at room temperature. Chemical shifts (δ) are reported in parts per million (ppm) and residual solvent peaks have been used as an internal reference. Peak multiplicities have been abbreviated as follows: s (singlet), d (doublet), dd (double-doublet), m (multiplet). [^{18}F]Fluoride was produced by a GE PETtrace cyclotron by 16 MeV irradiation of enriched [^{18}O]H_2O target, supplied by Alliance Medical Radiopharmacy Ltd (Warwick, UK). Automated radiosynthesies were performed using the GE FASTlab™ automated synthesis module (GE Healthcare Life Sciences, Amersham, UK). Solid phase extraction (SPE) cartridges were purchased from Waters (Elstree, Hertfordshire, UK) and used according to the manufacturers recommended guidelines. Semi-preparative RP-HPLC was performed using a Shimadzu LC20-AT pump attached to a custom-built system, equipped with an Agilent Eclipse XDB-C18, 5µ (250 x 9.4 mm) column using an isocratic mobile phase of MeCN (44%), H_2O (56%) and 0.1% H_3PO_4 (14.8 M) at a flow rate of 3 mL/min. Reaction efficiency and radioactive product identity was determined by RP-HPLC using an Agilent 1200 series instrument connected to a flow-ram detector (Lablogic, Sheffield, UK).

2.0 Synthesis

Compound **1** was prepared according to a reported literature procedure.[1]

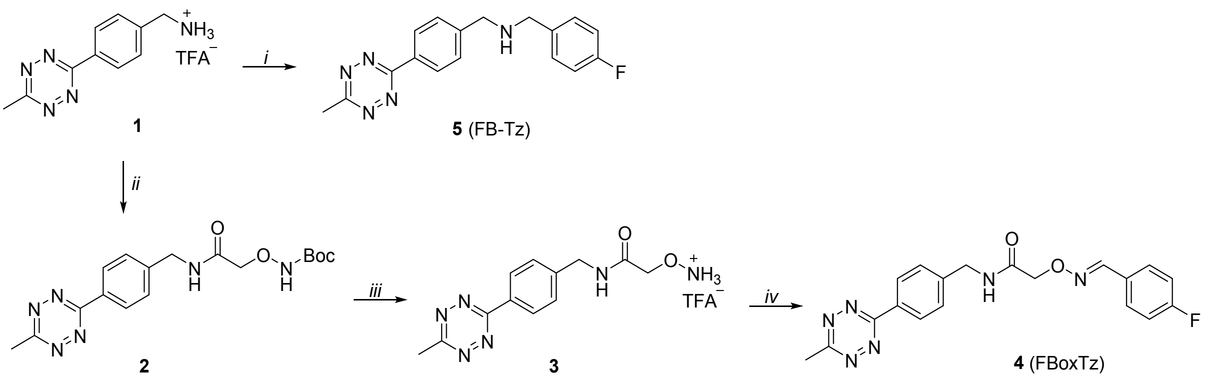

Scheme 1. Synthesis of radiochemistry precursors (**1** and **3**) and reference materials (**5** and **4**) for [^{18}F]FB-Tz and [^{18}F]FBoxTz, respectively. *Reaction conditions: i)* 4-fluorobenzalehyde, sodium triacetoxyborohyride, MeCN, RT, 16 h; *ii)* 2,5-dioxopyrrolidin-1-yl 2-(((tert-butoxycarbonyl)amino)oxy)acetate, triethylamine, MeCN, 2 h, RT*; iii)* TFA, DCM, 1 h, RT*; iv)* 4-fluorobenzaldehyde, MeCN, 16 h, RT.

Synthesis of (4-(6-methyl-1,2,4,5-tetrazin-3-yl)phenyl)methanaminium trifluoroacetate salt (1). Synthesised according to a reported literature procedure as a pink solid.[36] 1H-NMR (400 MHz, DMSO-d6) δ 8.64 – 8.47 (d, J = 8.3 Hz, 2H), 8.31 (s, 3H), 7.75 (d, J = 8.3 Hz, 2H), 4.20 (d, J = 5.1 Hz, 2H), 3.02 (s, 3H). 13C-NMR (400 MHz, DMSO-d6) δ 21.3, 42.5, 128.1, 130.2, 132.5, 138.8, 163.5, 167.8.

Synthesis of tert-butyl (2-((4-(6-methyl-1,2,4,5-tetrazin-3-yl)benzyl)amino)-2-oxoethoxy)carbamate (2). To a solution of (boc-aminooxy)acetic acid (500 mg, 2.6 mmol) in MeCN (6 mL) and pyridine (6 mL) under nitrogen was added N,N,N′,N′-tetramethyl-O-(N-succinimidyl)uronium tetrafluoroborate (TSTU, 1.50 g, 5.0 mmol). The reaction was stirred at ambient temperature for 16 h, after which the reaction mixture was evaporated to dryness. The residue was dissolved in DCM (20 mL) and washed with phosphoric acid (0.1 M, 3 × 10 mL) and brine (3 × 10 mL). The organic layer was dried over $MgSO_4$ before filtering. Solvent was removed in vacuo to yield 2,5-dioxopyrrolidin-1-yl 2-(((tert-butoxycarbonyl)amino)oxy)acetate, an off-white oil which was used immediately without further purification. To a flask containing 1 (397 mg, 1.3 mmol) in dry MeCN (20 mL) was added 2,5-dioxopyrrolidin-1-yl 2-(((tert-butoxycarbonyl)amino)oxy)acetate (605mg, 2.1

mmol) and triethylamine (1 mL). The reaction was stirred for 2 h and monitored by silica TLC (3:1 EtOAc/hexane). Solvent was removed in vacuo and the residue was purified by silica column chromatography (1:1 EtOAc/hexane). Pure fractions were combined and evaporated in vacuo to give a pink solid (410 mg, 82% yield). 1H-NMR (400 MHz, chloroform-d) δ 8.51 – 8.39 (m, 2H), 7.55 – 7.38 (m, 2H), 4.55 (d, J= 6.1 Hz, 2H), 4.34 (s, 2H), 3.02 (s, 3H), 1.35 (s, 9H). 13C-NMR (400 MHz, chloroform-d) δ 21.1, 28.0, 76.3, 83.4, 128.1, 128.4, 130.7, 143.1, 157.9, 163.9, 167.2, 169.0. ESI-MS (m/z): [M+H]+ calc'd for C17H23N6O4, 375.1781; found, 375.1776.

Synthesis of O-(2-((4-(6-methyl-1,2,4,5-tetrazin-3-yl)benzyl)amino)-2-oxoethyl)hydroxylammonium trifluoroacetate salt (3). To a flask containing 2 (350 mg, 0.9 mmol) was added DCM (1 mL) and trifluoroacetic acid (1 mL). The reaction was stirred for 16 h and monitored by silica TLC (1:1 EtOAc/hexane). Upon completion, solvent was removed in vacuo and the residue was precipitated from diethyl ether and filtered. The filtrate was washed with diethyl ether (3 × 10 mL) and dried to give the title compound (267 mg, 80% yield). 1H-NMR (400 MHz, DMSO-d6) δ 8.44 (d, J= 8.4 Hz, 2H), 7.67 – 7.52 (m, 2H), 4.84 (d, J= 6.0 Hz, 2H), 4.44 (s, 2H), 3.00 (s, 3H). 13C-NMR (400 MHz, DMSO-d6) δ 21.3, 42.1, 72.8, 127.9, 128.6, 131.0, 144.2, 163.6, 167.6, 168.4. ESI-MS (m/z): [M+H]+ calc'd for C12H15N6O2, 275.1256; found, 275.1253.

Synthesis of (E)-2-(((4-fluorobenzylidene)amino)oxy)-N-(4-(6-methyl-1,2,4,5-tetrazin-3-yl)benzyl)acetamide (4). To a flask containing 3 (50 mg, 0.13 mmol) in MeCN (1 mL) was added 4-fluorobenzaldehyde (24 mg, 0.19 mmol). The reaction was stirred at room temperature for 1 h and monitored by silica TLC (3:1 EtOAc/hexane). Upon complete reaction, solvent was removed in vacuo and the product was precipitated from EtOAc and hexane to yield a pink solid (28 mg, 56 %). 1H-NMR (400 MHz, chloroform-d) δ 8.57 – 8.50 (m, 2H), 8.19 (s, 1H), 7.66 – 7.57 (m, 2H), 7.55 – 7.49 (m, 2H), 7.10 (t, J = 8.7 Hz, 2H), 6.68 (s, 1H), 4.76 (s, 2H), 4.68 (d, J = 6.2 Hz, 2H), 3.12 (s, 3H). 13C-NMR (400 MHz, chloroform-d) δ 21.2, 42.6, 73.3, 116.2, 127.2, 128.2, 128.3, 129.2, 129.3, 131.0, 142.8, 150.0 163.8, 167.3, 169.7. ESI-MS (m/z): [M+H]+ calc'd for C19H18N6O2F, 381.1475; found, 381.1474.

Synthesis of N-(4-fluorobenzyl)-1-(4-(6-methyl-1,2,4,5-tetrazin-3-yl)phenyl)methanamine (5). (4-(6-methyl-1,2,4,5-tetrazin-3-yl)phenyl)methanaminium trifluoroacetate salt (200 mg, 0.66 mmol) and 4-fluorobenzalehyde (81 mg, 0.66 mmol) was

added to dry MeCN followed by the addition of sodium triacetoxyborohyride (212 mg, 1.00 mmol). The reaction was stirred for 16 h at ambient temperature. Solvent was removed in vacuo and the residue was purified by column chromatography (1:1 EtOAc/hexane, silica) to yield the product as a pink solid (71 mg, 35 %). ^{1}H-NMR (400 MHz, chloroform-d) δ 8.54 – 8.43 (m, 2H), 7.55 – 7.47 (m, 2H), 7.30 – 7.22 (m, 2H), 7.00 – 7.22 (m, 2H), 7.00 – 6.90 (m, 2H), 3.84 (s, 2H), 3.75 (s, 2H), 3.02 (s, 3H). ^{13}C-NMR (400 MHz, chloroform-d) δ 21.1, 52.7, 115.4, 128.06, 128.9, 129.8, 130.6, 135.6, 145.1, 160.8, 163.2, 164.0, 167.2. ^{19}F-NMR (376 MHz, chloroform-d) δ -115.77. ESI-MS (m/z): $[M+H]^+$ calc'd for $C_{17}H_{17}N_5F$, 310.1468; found, 310.1464.

2.1 NMR Spectra

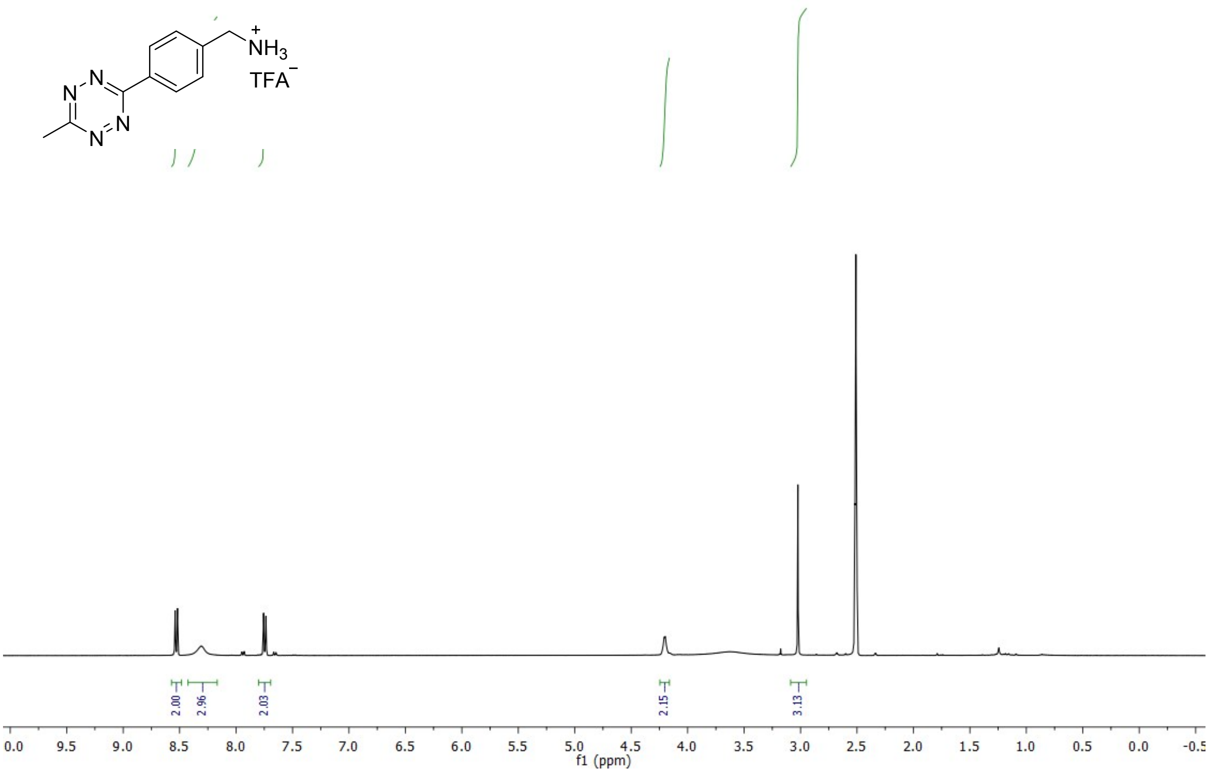

Figure 1. ^{1}H-NMR spectra for (4-(6-methyl-1,2,4,5-tetrazin-3-yl)phenyl)methanaminium trifluoroacetic acid salt (**1**)

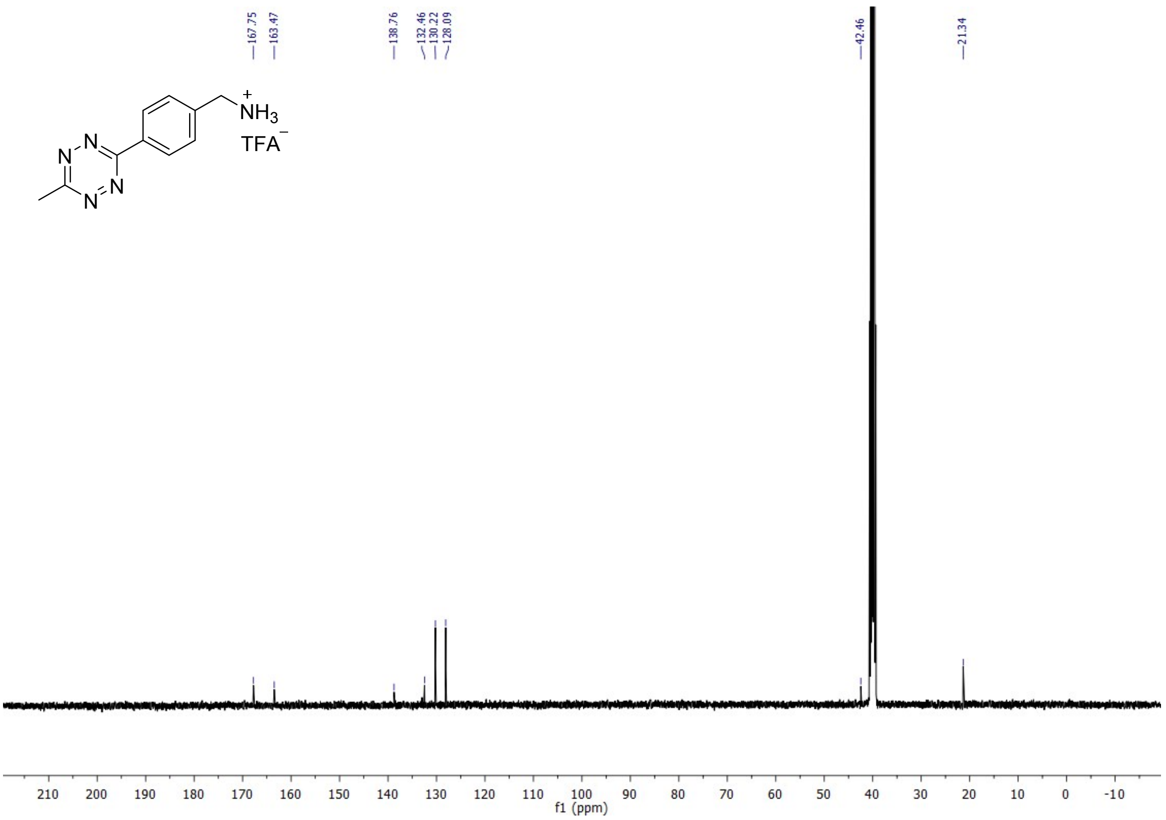

Figure 2. ^{13}C-NMR spectra for (4-(6-methyl-1,2,4,5-tetrazin-3-yl)phenyl)methanaminium trifluoroacetic acid salt (**1**)

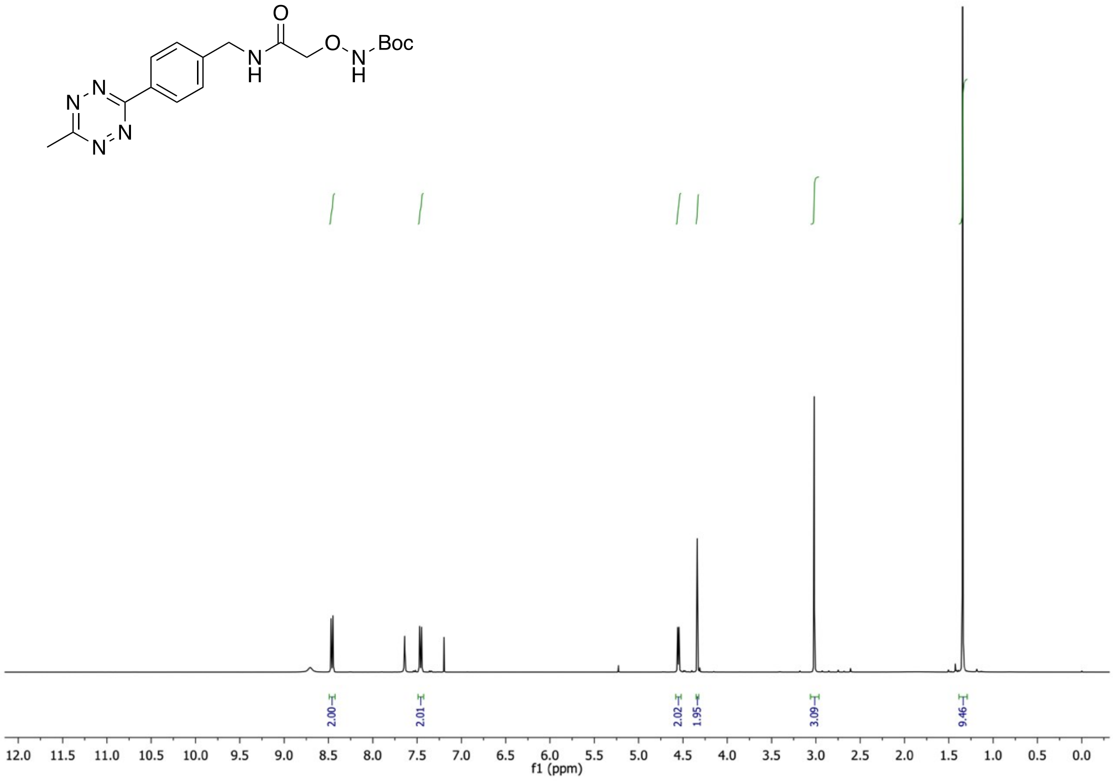

Figure 3. ^{1}H-NMR spectra for tert-butyl (2-((4-(6-methyl-1,2,4,5-tetrazin-3-yl)benzyl)amino)-2-oxoethoxy)carbamate (**2**)

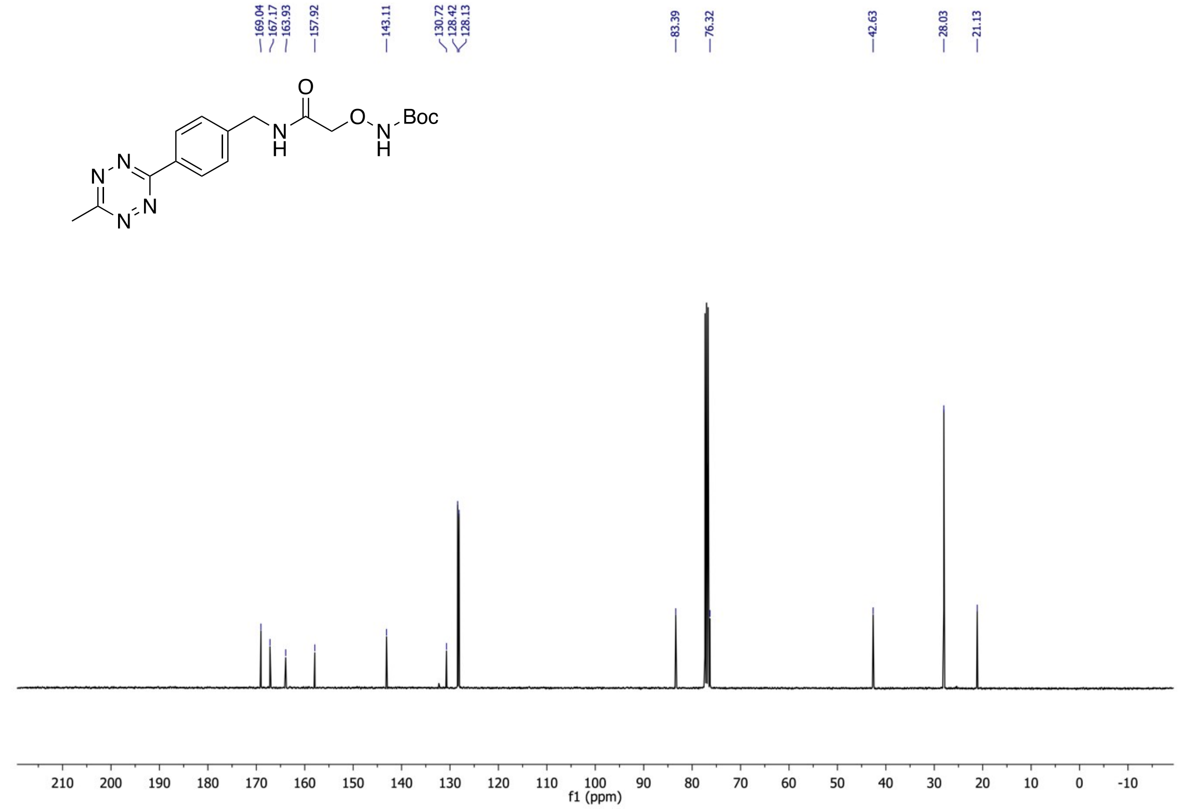

Figure 4. ^{13}C-NMR spectra for tert-butyl (2-((4-(6-methyl-1,2,4,5-tetrazin-3-yl)benzyl)amino)-2-oxoethoxy)carbamate (**2**)

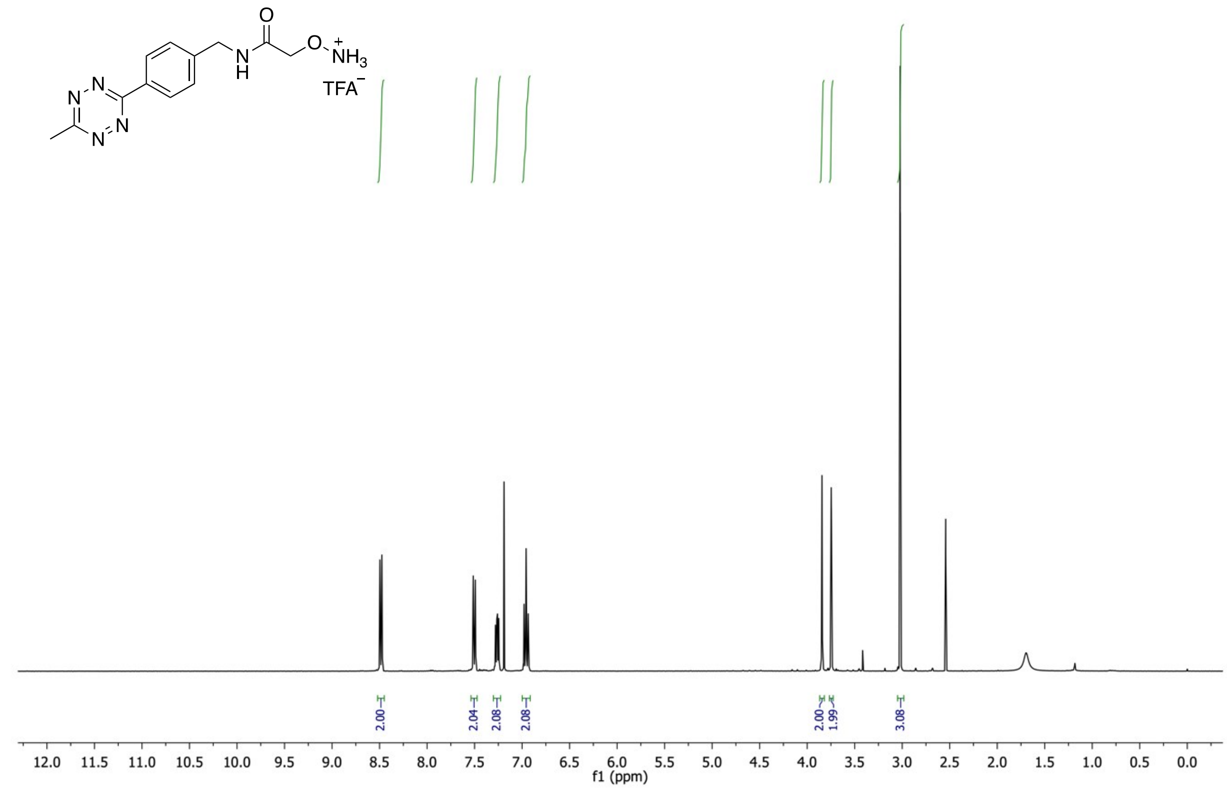

Figure 5. ^{1}H-NMR spectra for O-(2-((4-(6-methyl-1,2,4,5-tetrazin-3-yl)benzyl)amino)-2-oxoethyl)hydroxylammonium trifluoroacetic acid salt (**3**)

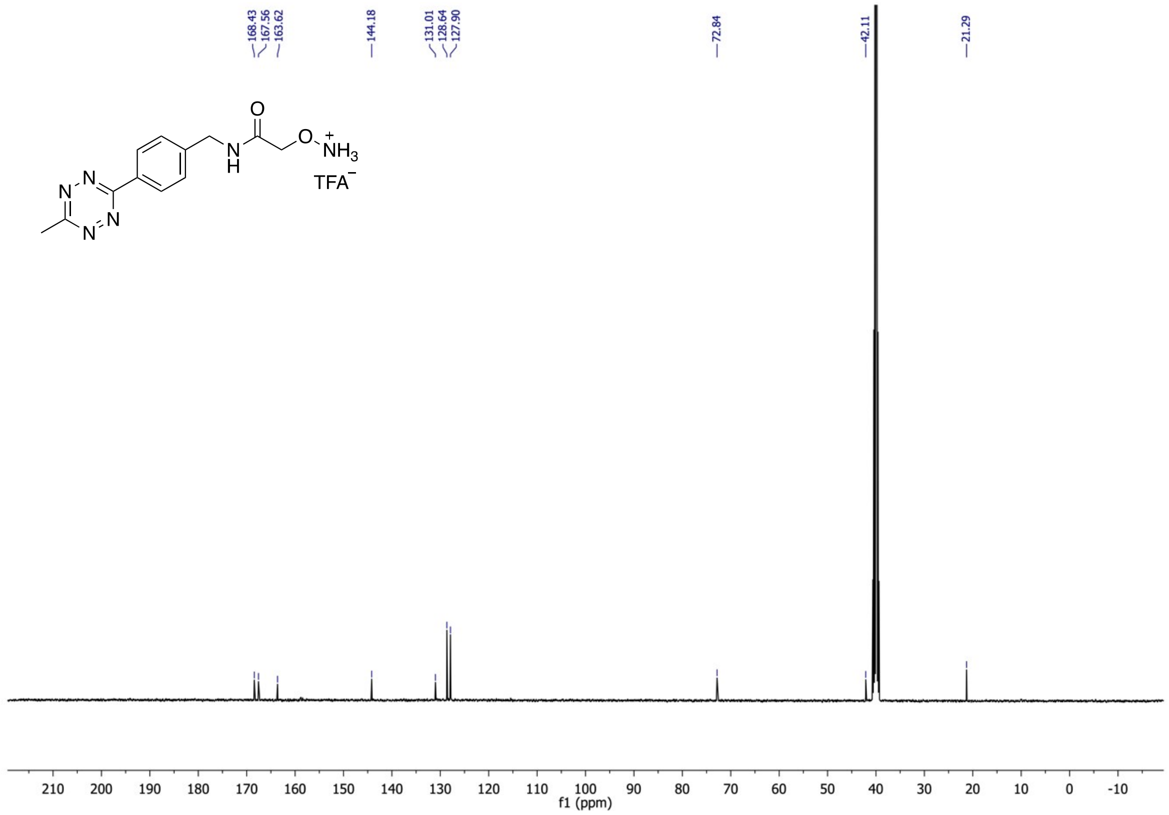

Figure 6. ^{13}C-NMR spectra for O-(2-((4-(6-methyl-1,2,4,5-tetrazin-3-yl)benzyl)amino)-2-oxoethyl)hydroxylammonium trifluoroacetic acid salt (**3**)

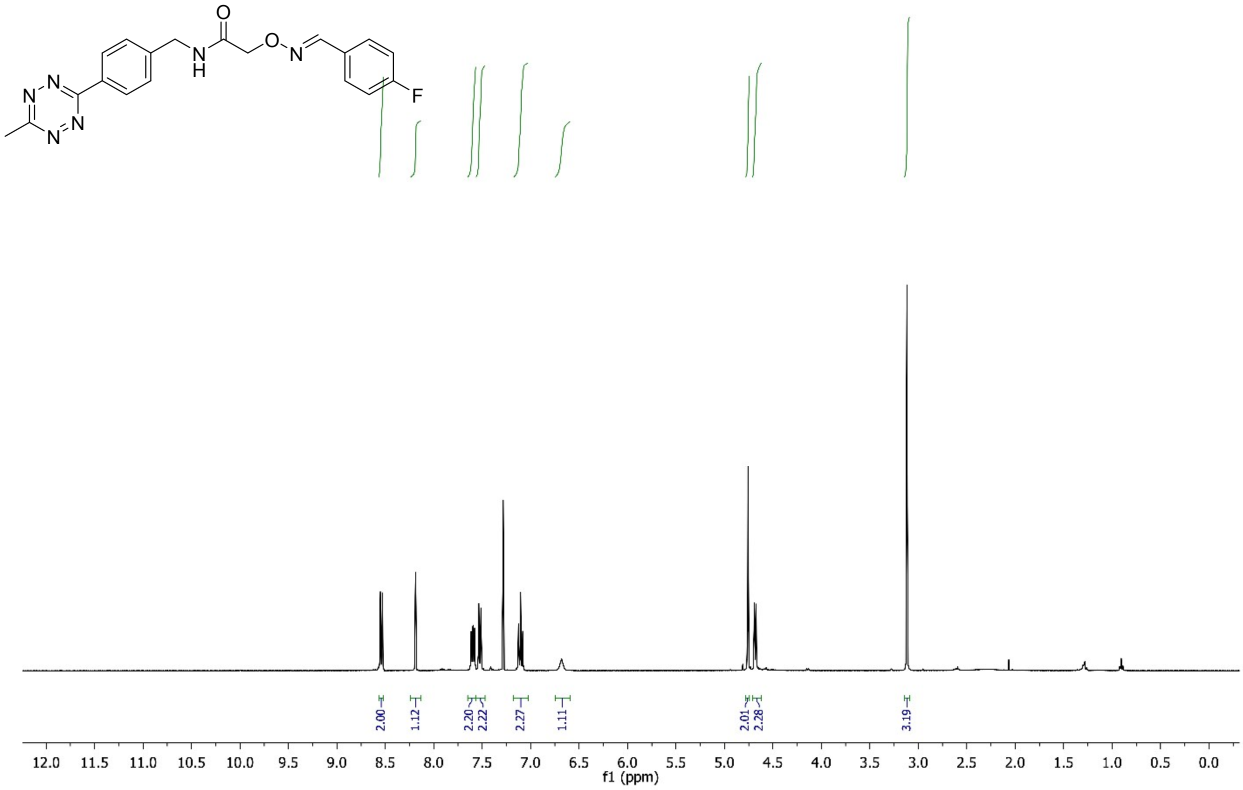

Figure 7. ^{1}H-NMR spectra for (E)-2-(((4-fluorobenzylidene)amino)oxy)-N-(4-(6-methyl-1,2,4,5-tetrazin-3-yl)benzyl)acetamide (**4**)

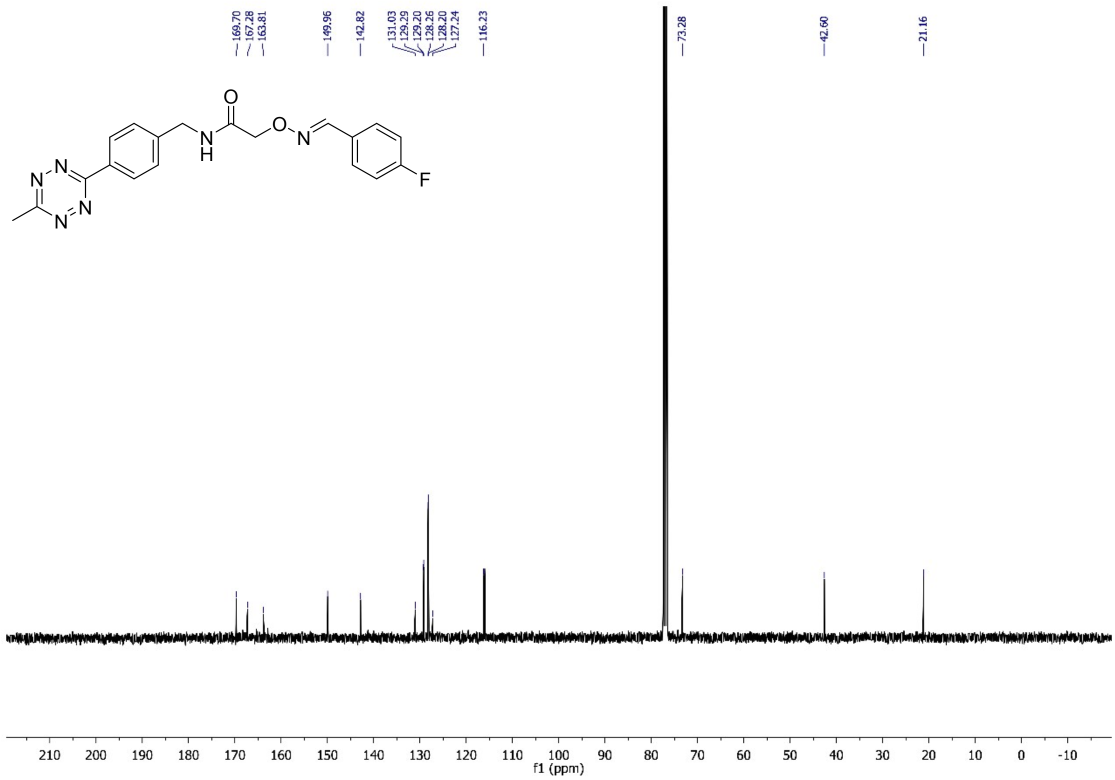

Figure 8. ^{13}C-NMR spectra for (E)-2-(((4-fluorobenzylidene)amino)oxy)-N-(4-(6-methyl-1,2,4,5-tetrazin-3-yl)benzyl)acetamide (**4**)

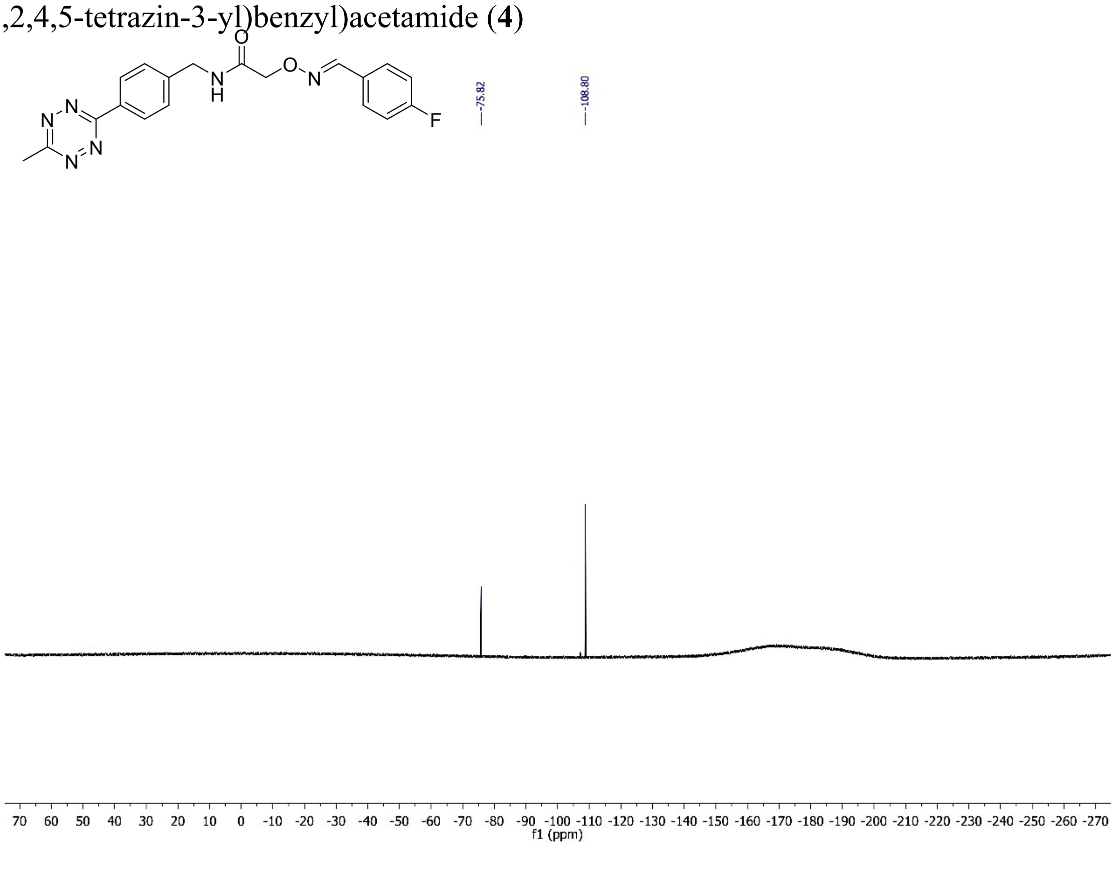

Figure 9. ^{19}F-NMR spectra for (E)-2-(((4-fluorobenzylidene)amino)oxy)-N-(4-(6-methyl-1,2,4,5-tetrazin-3-yl)benzyl)acetamide (**4**)

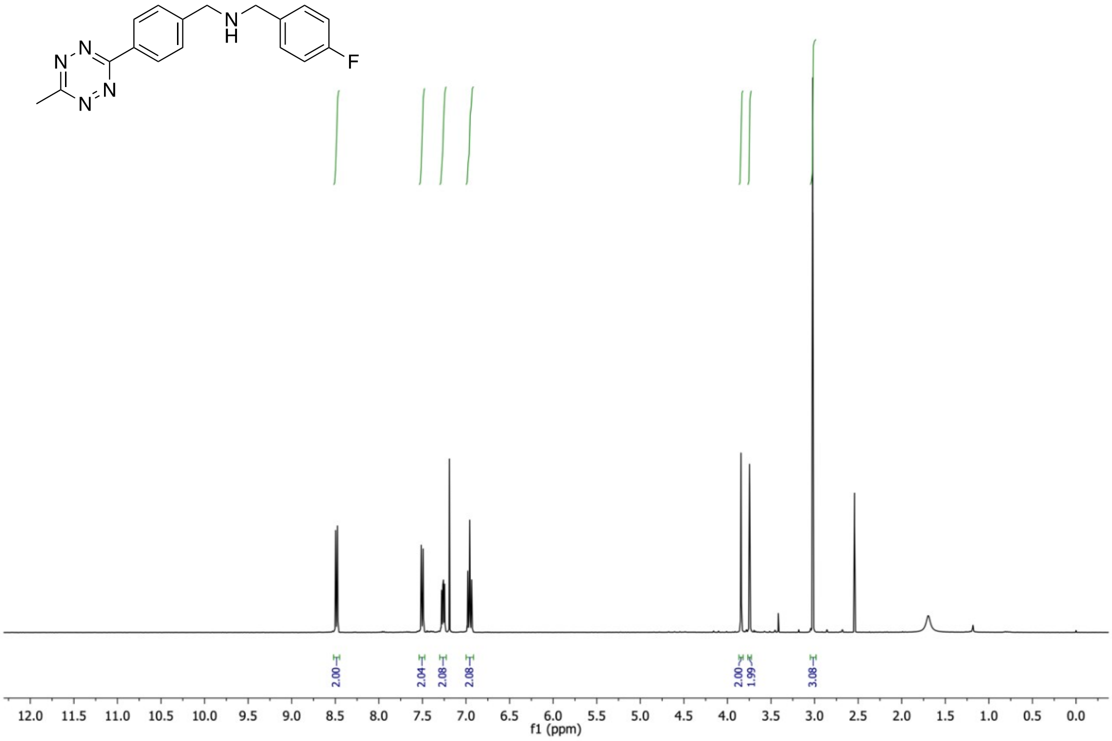

Figure 10. ^{1}H-NMR spectra for N-(4-fluorobenzyl)-1-(4-(6-methyl-1,2,4,5-tetrazin-3-yl)phenyl)methanamine (**5**)

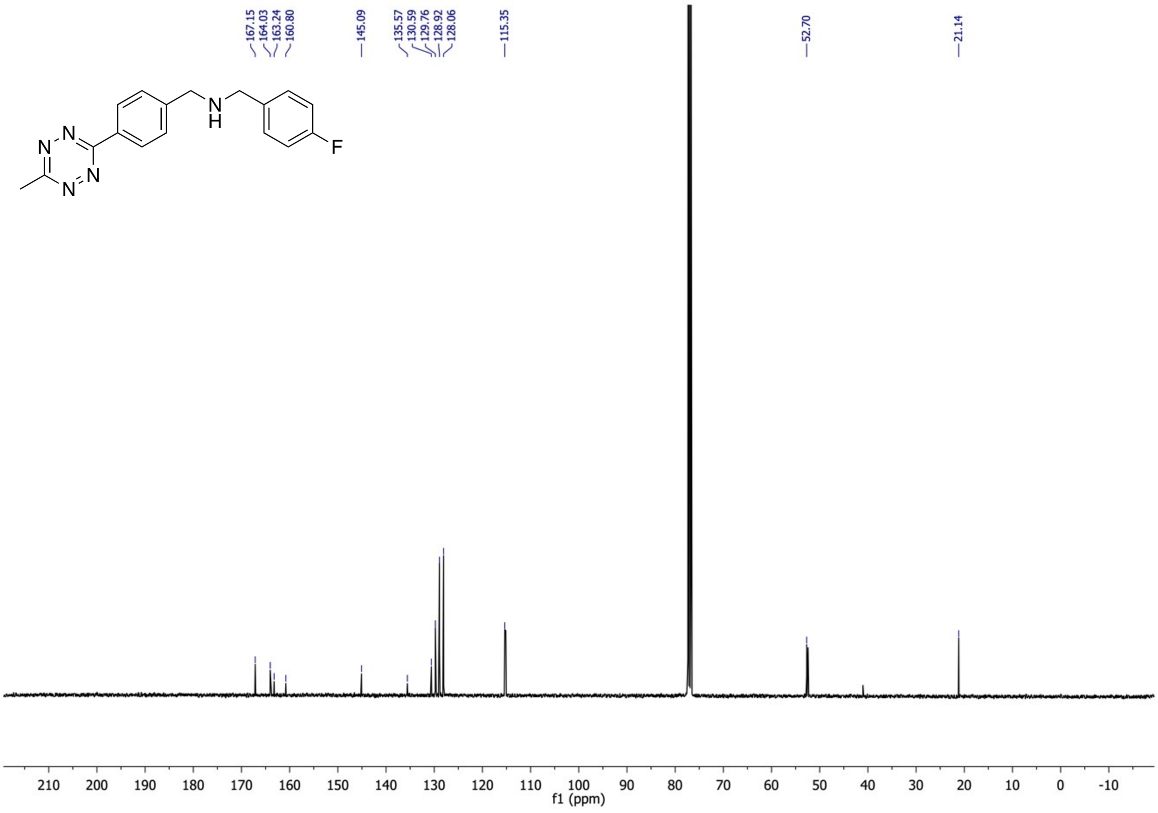

Figure 11. ^{13}C-NMR spectra for N-(4-fluorobenzyl)-1-(4-(6-methyl-1,2,4,5-tetrazin-3-yl)phenyl)methanamine (**5**)

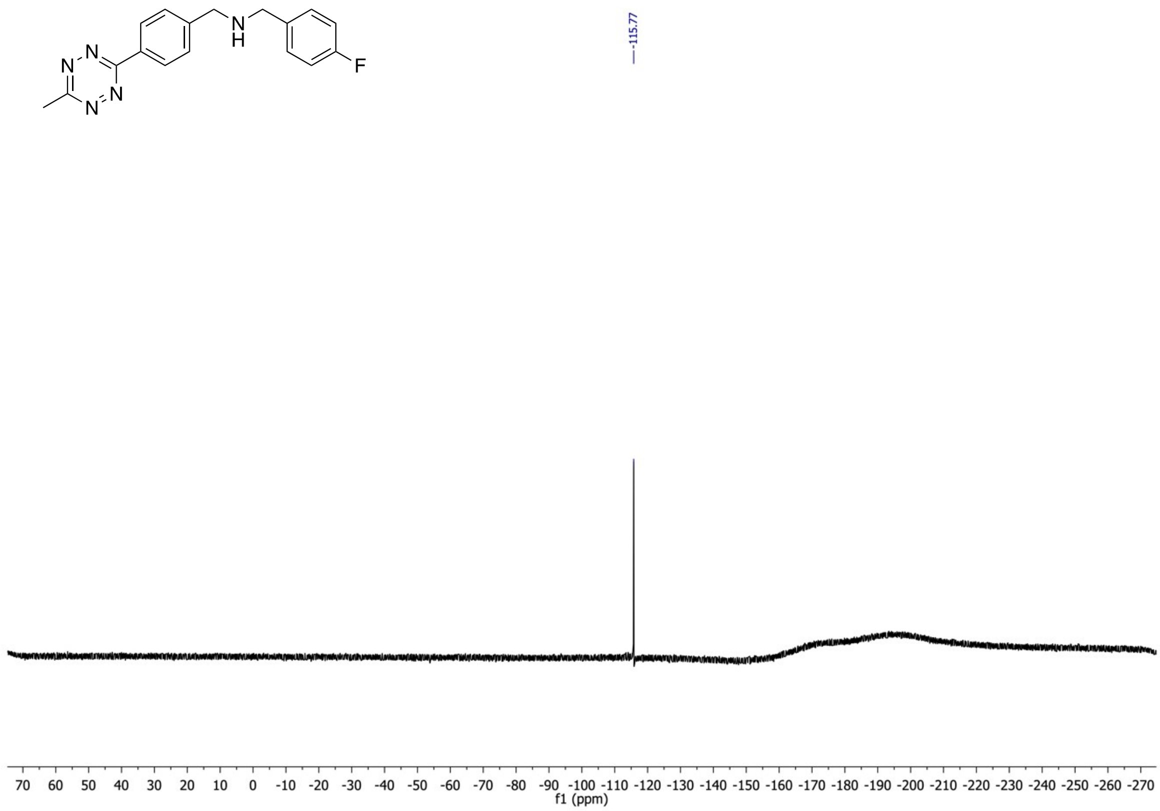

Figure 12. ^{19}F-NMR spectra for N-(4-fluorobenzyl)-1-(4-(6-methyl-1,2,4,5-tetrazin-3-yl)phenyl)methanamine (**5**)

3.0 Determining the number of TCO moieties per molecule of IL2

To a vial of TCO-PEG$_4$-IL2 (200 µg, 72 µL, 12.66 nmol) was added a 10-fold excess of 6-methyl-tetrazine-5-FAM (126.6 nmol) in MeOH (10 µL). The reaction was shaken in the dark for 1 h. A Zeba™ Spin Desalting Column (7 kDa MWCO, 0.5 mL) was equilibrated in PBS containing SDS (0.05% w/v) and the reaction mixture was loaded onto the column for purification. The resulting solution was analysed by UV-vis spectroscopy (nanodrop) to determine the dye/protein ratio. The experiment was performed in triplicate for two separately synthesised batches of TCO-PEG$_4$-IL2. The molar absorptivity coefficient (ε) for TCO-PEG$_4$-IL2 was determined experimentally. The protein quantity of a stock solution of TCO-PEG$_4$-IL2 was determined by BCA assay from which a series of standard solutions were prepared (2.6, 1.3, 0.65 and 0.065 µg/uL). Each sample was analysed in triplicate using UV-vis spectroscopy (nanodrop) for absorbance at 280 nm. A plot of absorbance (A280) and concentration (M) was created to calculate ε = slope.

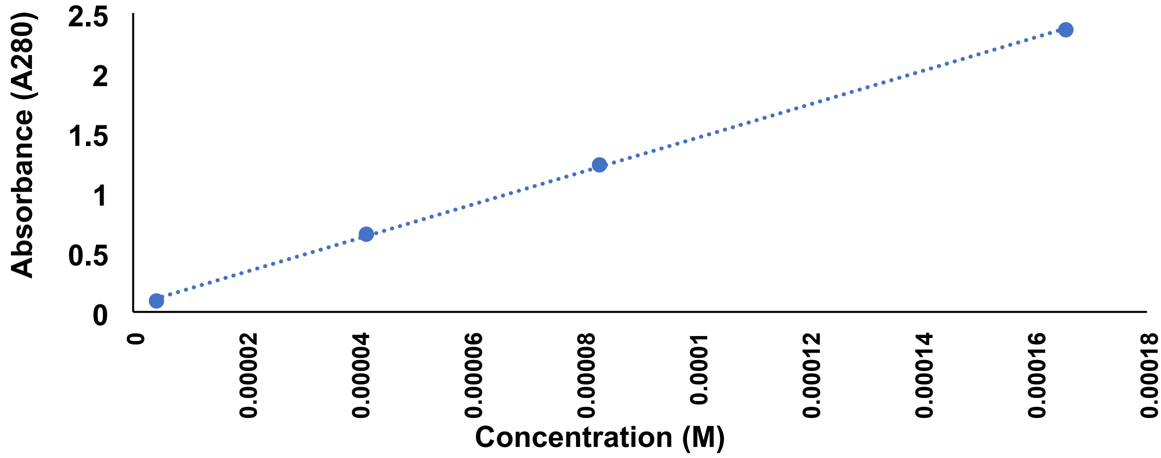

Figure 13. A plot of TCO-PEG4-IL2 concentration against absorbance to determine the molar extinction coefficient (ε) for protein quantification.

The number of TCO moieties per molecule of TCO-PEG$_4$-IL2 was determined by UV-vis spectroscopy using a fluorescent tetrazine dye (6-methyl-tetrazine-5-FAM) in a 10-fold molar excess with respect to TCO-PEG$_4$-IL2. The absorption at 280 nm (A_{280}) and 492 nm (A_{492}) was measured using a nanodrop UV-vis spectrometer.

The following equations were used to determine the dye:protein ratio:

$$Protein\ Conc\ (M) = \frac{A_{280} - (A_{492} \times 0.3)}{13911\ M^{-1}cm^{-1}} \times 10$$

$$Dye{:}Protein = \frac{A_{494}}{68000M^{-1}cm^{-1} \times Protein\ Conc\ (M)} \times 10$$

0.3 = correction factor for FITC dye

10 = dilution factor (each purified sample was diluted 10-fold to avoid detector saturation)

13911 $M^{-1}cm^{-1}$ = experimentally determined molar absorptivity of TCO-PEG_4-IL2

68000 $M^{-1}cm^{-1}$ = molar absorptivity of FITC

4.0 Radiosynthesis

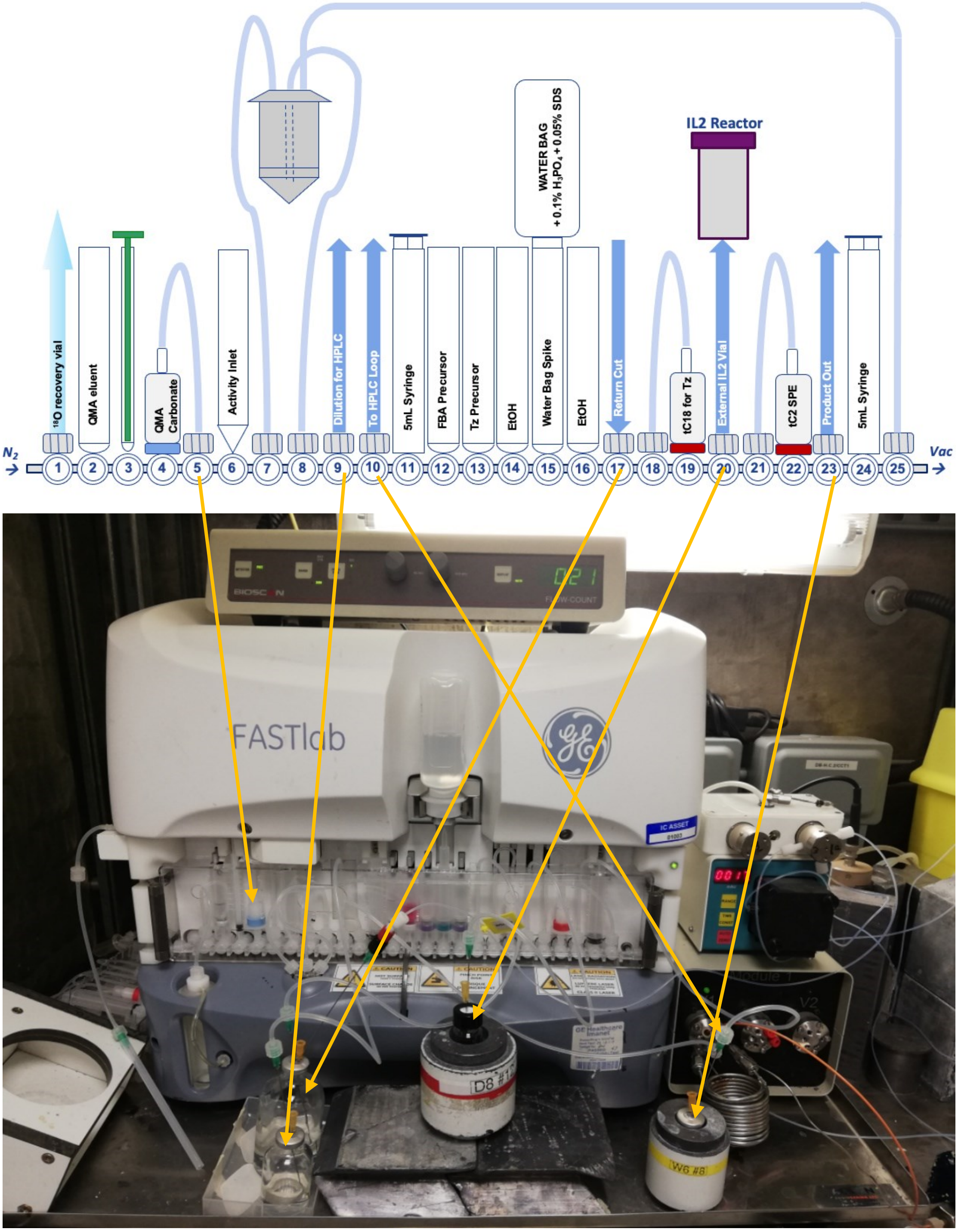

Figure 14. Schematic and photograph of the FASTLab™ cassette setup for the automated radiosynthesis of [^{18}F]TTCO-IL2.

Table 1. Reagents/consumable positions on the FASTLab™ cassette shown in figure 14.

Position	Description	Contents
2	QMA Eluent	$KHCO_3$ (3.5 mg/mL, 200 µL)<br>K_{222} (6 mg/mL, 800 µL)
12	FBA Precursor	FBA precursor (3 mg in 1.4 mL MeCN)
13	Tetrazine Precursor	Aminooxy tetrazine (8 mg in 1 mL MeCN)<br>Aniline HCl (6 mg in 400 µL H_2O)
14	Ethanol	100% EtOH (4 mL)
15	Water Bag	0.05% SDS (50 mg/100 mL)<br>0.1% H_3PO_4 (100 µL/100 mL)
16	Ethanol	100% EtOH (4 mL)
20	IL2 Reactor<br>(10 mL Wheaton)	TCO-PEG$_4$-IL2 (200 µg) in PBS + 0.05% SDS

Bulk solutions:

Position	Description	Contents
9	Loop dilution	H_2O + 0.1% H_3PO_4 (7.5 mL)
17	Return cut peak	H_2O + 0.1% H_3PO_4 (35 mL)
23	Product vial	PBS + 0.05% SDS + 4.5% Glucose (9 mL)

SPE cartridges:

Position	Description
4 - 5	QMA Bicarb
18 - 19	tC18 plus [conditioned with EtOH (5 mL) and H_2O (5 mL)]
21 - 22	tC2 plus [conditioned with EtOH (5 mL) and H_2O (5 mL)]

Procedure:

Fluoride drying: [^{18}F]fluoride in ^{18}O-water was drawn by vacuum onto the cassette through the activity inlet (position 6). The [^{18}F]fluoride was trapped on a QMA cartridge (position 4) and eluted into the reactor using the eluent QMA eluent mixture (position 2) with the 1 mL syringe (position 3). The [^{18}F]fluoride was dried at 120 °C for 7 min, followed by 70 °C for 5 min.

Synthesis of [^{18}F]FBoxTz: The [^{18}F]FBA precursor in MeCN was added to the reactor by pressurising the vial and heated to 90 °C for 7 min, after which the reactor was allowed to cool to 40 °C for 6 min. To the reaction mixture was added the [^{18}F]FBoxTz precursor by syringe (position 11) and heated at 40 °C for 10 min. To purify the resulting [^{18}F]FBoxTz, the contents of the reactor were evacuated under positive pressure to an external reaction vial (position 9). The reactor was rinsed with water (2 mL) using the syringe (position 11) and evacuated to the external dilution vial (position 9). The contents of the dilution vial (position 9) were loaded onto the 10 mL HPLC loop (position 10) using the syringe (position 11). The desired peak was cut into a dilution vial from the external semi-preparative HPLC system and returned to the cassette (position 17) and trapped on a tC18 SPE cartridge (position 19) using the syringe (position 11).

Synthesis of [^{18}F]TTCO-IL2: The trapped [^{18}F]FBoxTz was eluted from the tC18 SPE cartridge with EtOH (position 14) using a syringe (position 11) into the external Wheaton vial (10 mL) which contained a solution of TCO-PEG$_4$-IL2 (position 20). The IEDDA reaction proceeded at ambient temperature for 15 min, before being diluted with 9 mL from the water bag (position 15). The diluted mixture was loaded through a tC2 SPE cartridge (position 21). A 50% EtOH/Water bag mixture was made inside a 5 mL syringe (position 24) by mixing the EtOH (position 14) and water bag (position 15) which was used to wash the tC2 SPE cartridge (position 22), removing small molecule components of the reaction (i.e. unreacted [^{18}F]FBoxTz). The desired [^{18}F]TTCO-IL2 was eluted from the tC2 SPE cartridge in 1 mL EtOH (position 16) using a syringe (position 24) into a product vial (position 23) containing reformulation buffer (9 mL).

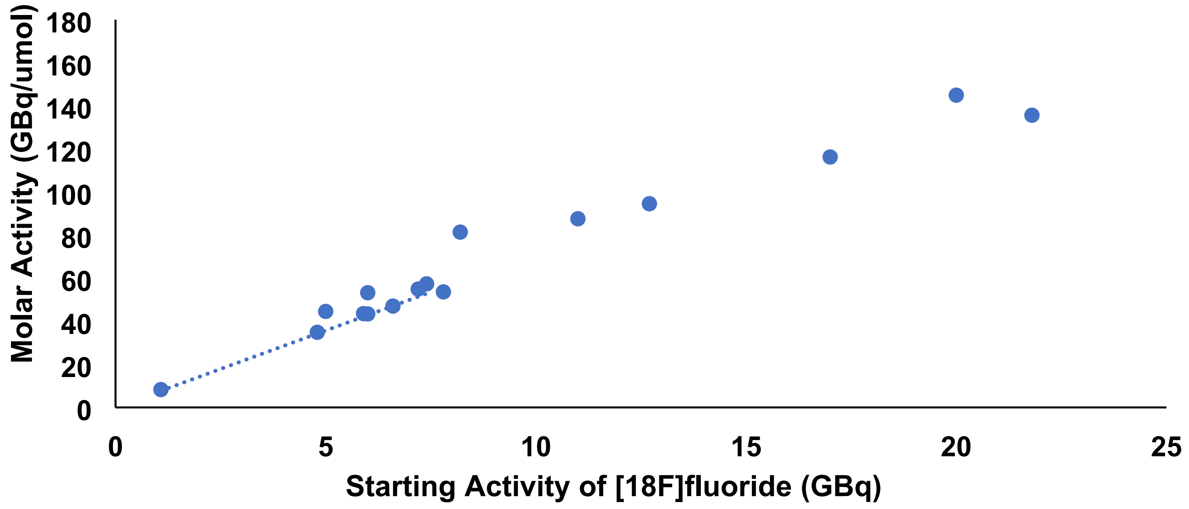

Figure 15. A line graph showing the relationship between starting activities of [^{18}F]fluoride and molar activity (n = 16).

5.0 HPLC and TLC Chromatograms

Semi-preparative HPLC

**Column:**	Agilent XDB-C18
**Mobile Phase:**	Isocratic<br>44% MeCN<br>56% H_2O<br>+ 0.1%H_3PO_4
**Flow rate:**	3 mL/min

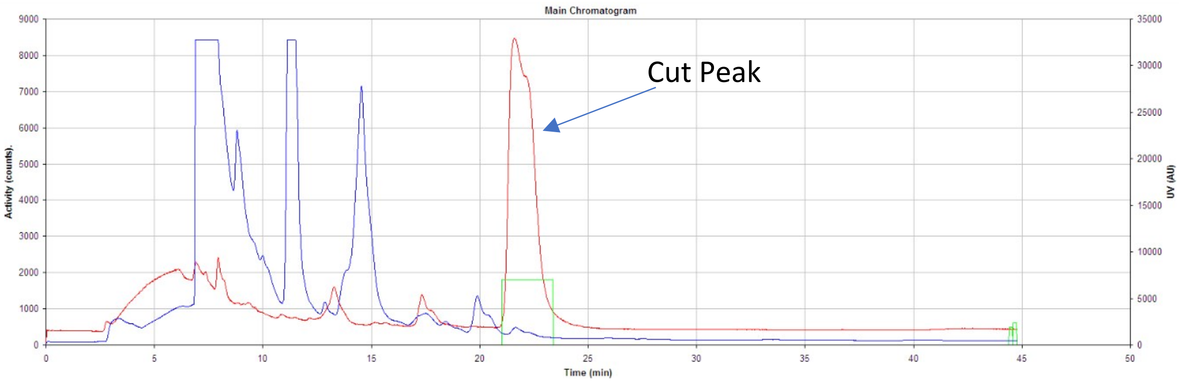

Figure 16. Represenative semi-preparative HPLC chromatogram showing the cut peak for the [^{18}F]FBoxTz prosthetic group. Blue line = UV, red line = radioactive counts.

Radio-TLC

TLC plate: iTLC-SG (Glass microfiber chromatography paper impregnated with silica gel) Cat no. SGI0001

Mobile phase: Ethyl acetate / hexane (2:1)

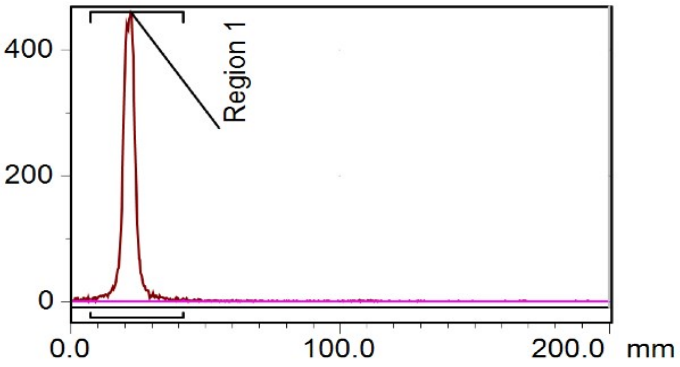

Figure 17. Representative radio-TLC chromatogram showing purified [^{18}F]TTCO-IL2 (R_f= 0.0 and [^{18}F]FBoxTz R_f = 1.0)

<u>**Analytical HPLC**</u>

Column: Phenomenex Aeris 3.6 µm WIDEPORE 200Å, 150 x 4.6 mm (S/N: H19-302540)

Mobile Phase: Gradient
A: H_2O + 0.1% TFA, B: MeCN

0 min – A:95%
1 min – A:95%
8 min – A:5%
20 min – A:5%

Flow rate: 1 mL/min

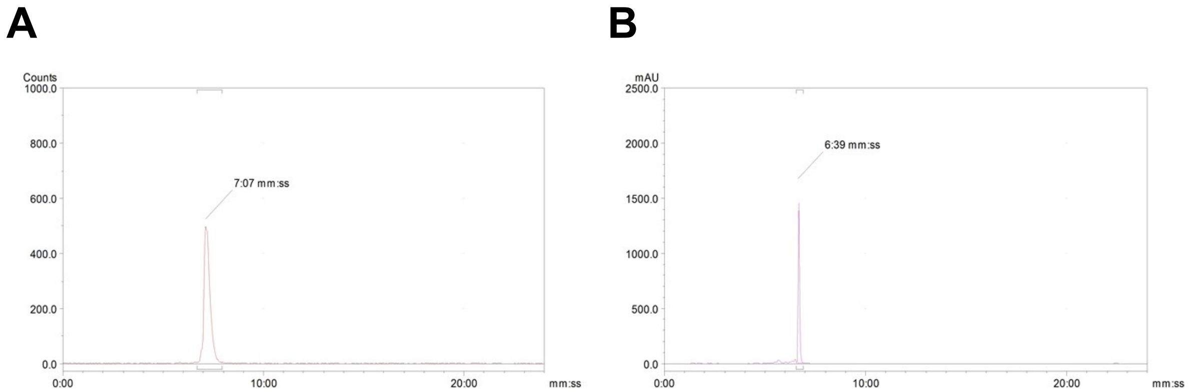

Figure 18. Representative analytical chromatograms showing **A**) radioactive [^{18}F]FBoxTz (t_R = 7:07 mm:ss) and **B**) reference standard **4** (t_R = 6:39 mm:ss UV detector: 254 nm).

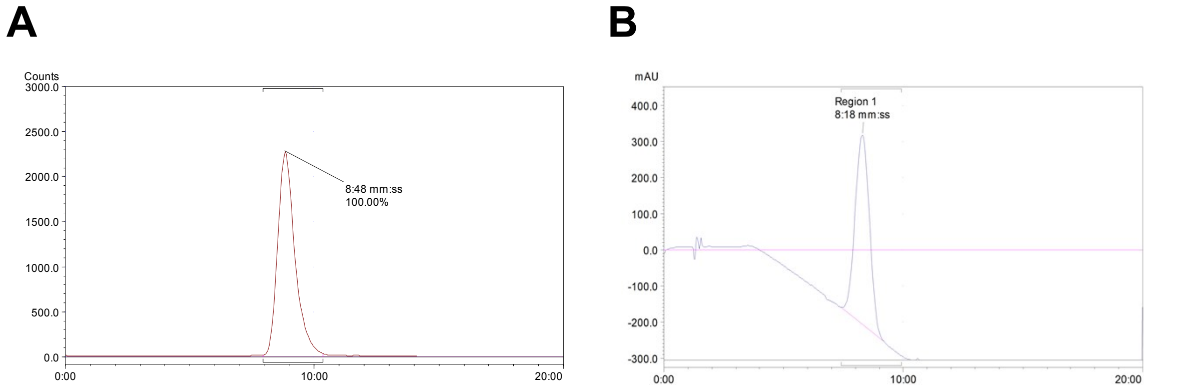

Figure 19. Representative analytical chromatograms showing **A**) radioactive [^{18}F]TTCO-IL2 (t_R = 8:48 mm:ss) and **B**) spiked IL2-PEG4-TCO (t_R = 8:18 mm:ss) as a reference standard (UV detector: 210 nm).

6.0 Radiostability assays

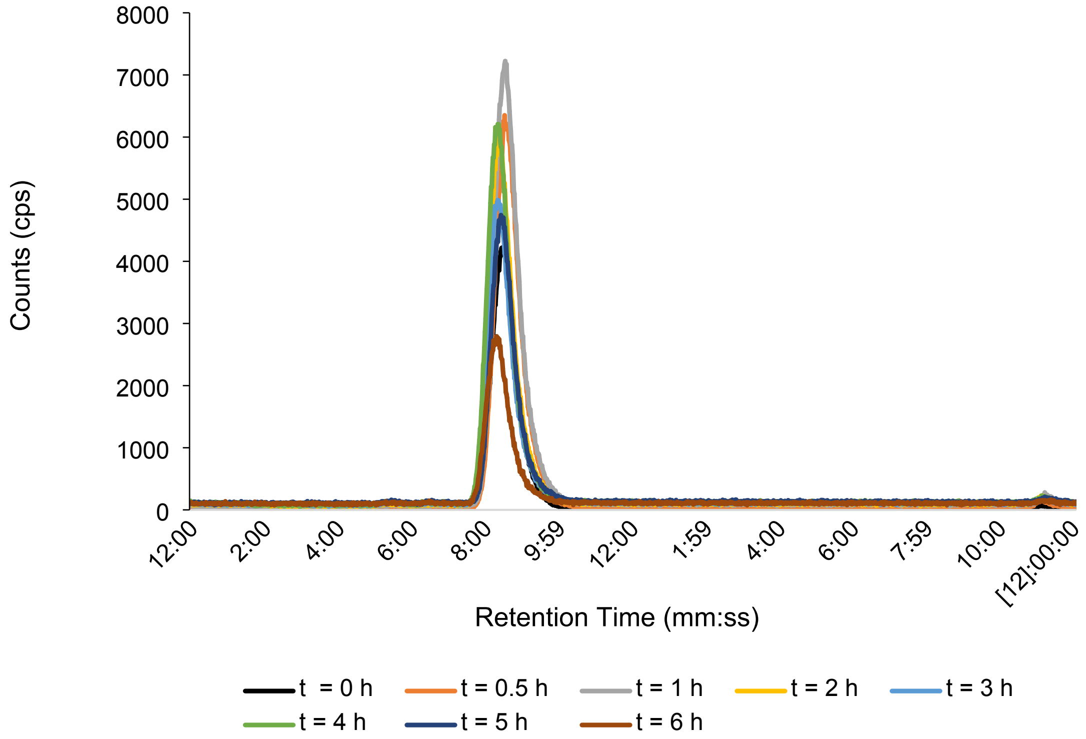

Figure 20. Radio-HPLC chromatograms showing the radiochemical stability of [^{18}F]TTCO-IL2 (t_R= 08:35 mm:ss) over 6 h.

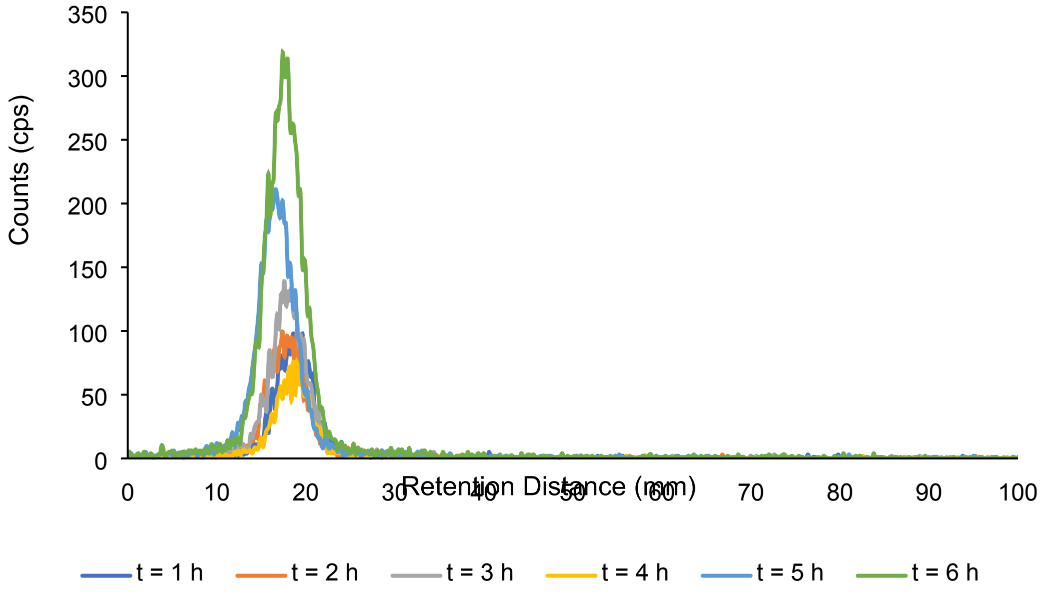

Figure 21. Radio-TLC chromatograms showing the radiochemical stability of [^{18}F]TTCO-IL2 (R_f = 0.0) over 6 h.

7.0 *In vitro* Metabolite analysis

Radioligand [^{18}F]TTCO-IL2 (4 MBq, ca. 100 µL), human liver microsomes (50 µL, 1 mg/mL) or mouse liver S9 (50 µL, 20 mg/mL), NADPH regeneration system A (50 µL), NADPH regeneration system B (10 µL) and PBS (400 µL, 0.1 M) were added to a plastic 1.5 mL tube. The mixture was incubated at 37 °C for 60 min and transferred into a plastic centrifuge tube (10 mL). Proteins were precipitated with ice-cold MeCN (2 mL) and the mixture was centrifuged (12,000 g, 3 min) to pellet the precipitated protein. The supernatant was removed and filtered (0.22 µm) before dilution in H_2O + 0.1% TFA (1:10) for HPLC analysis using an Agilent 1100 system with an in-line posiRAM metabolite detector (Lablogic, Sheffield, UK). The experiment was performed in n = 3 determinations and extraction efficiency from the protein pellet was calculated to be 19.5 ± 1.5 % from HLM and 27.5 ± 3.3% from mouse liver S9 fraction. Representative HPLC chromatograms are shown (Figure S22).

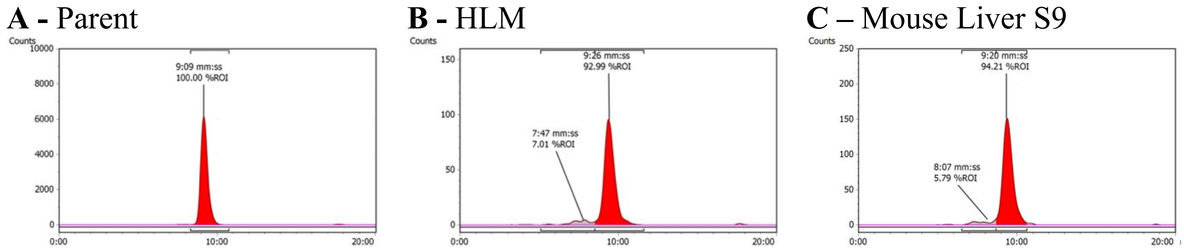

Figure 22. Representative radio-HPLC chromatograms for the *in vitro* metabolite assay of parent **A)** [^{18}F]TTCO-IL2 (t_R = 09:09 mm:ss) in **B)** human liver microsomes (HLM) and **C)** mouse S9. The extraction efficiency was determined to be 19.5 ± 1.5% for HLM and 27.5 ± 3.3% for mouse liver S9 (n = 3).

8.0 Cell culture

Prostate Cancer cells, PC3, were gifted by Professor Bevan (Imperial College London). Cells were maintained in RPMI-1640 media (Sigma Aldrich, Gillingham, UK) supplemented with 10% fetal calf serum (Sigma Aldrich), 1% L-glutamine, and 2% penicillin/streptomycin (Sigma Aldrich). NK-92 cells (immortalised Natural Killer cells) were purchased from ATCC, UK, and maintained in alpha MEM without ribonucleosides and deoxyribonucleosides with 2mM L-glutamine and 1.5 g/L sodium bicarbonate (Gibco), 0.2 mM myo-inositol (Sigma-Aldrich), 0.1 mM 2-mercaptoethanol (Gibco), 0.02 mM folic acid (Sigma Aldrich), 12.5% fetal calf serum (Sigma Aldrich) and 12.5% horse serum (ATCC), and 100 U/mL recombinant human interleukin-2 (Proleukin, Novartis). Peripheral blood mononuclear cells (PBMCs) were isolated from whole blood samples obtained from healthy volunteers under the approval of the West London Research Ethics Committee (Reference 10/H0707/7 and 12/WA/0196). PBMCs were isolated by Histopaque (Sigma Aldrich) separation. PBMCs were treated with 10 µg/mL phytohemagglutinin PHA-P (Sigma Aldrich) for 72 hours in the presence of 100 U/mL recombinant human interleukin-2 (Proleukin, Novartis). Human primary T cells were cultured in RPMI-1640 media (Sigma-Aldrich) with 10% Human AP Serum (Sigma Aldrich), 1% L-glutamine, and 2% penicillin/streptomycin (Sigma Aldrich). All cell lines were cultured at 37 °C and 5% CO_2. Receptor expression was determined by flow cytometry and the experimental procedure is described in the ESI.

9.0 Flow cytometry

Cells were collected and centrifuged at 1500 rpm for 5 min. Cell pellets were washed twice with cell staining buffer (BioLegend) and resuspended in 20 µL cell staining buffer containing FITC anti-CD3 (BioLegend,317306, Mouse IgG2a), and APC anti-human CD25 (BioLegend, 302610, Mouse IgG1) or APC anti-human CD132 (BioLegend, 338607, Rat IgG2b). After 30 minutes incubation at 4 °C, cells were washed twice and fixed with 1% paraformaldehyde. For CD122 staining, following incubation with primary antibody (R&D Systems, MAB224-100, Mouse IgG1), cells were washed twice and incubated with a rat anti-mouse FITC-conjugated secondary antibody (BioLegend, 406001, IgG) for 30 minutes at 4 °C prior to washing and fixing. For intracellular phosphorylated-STAT5 (Tyr694) staining, fixed cells wear permeabilised with 1x intracellular staining perm wash buffer (BioLegend, 421002) at 350xg for 7 minutes. After incubation with rabbit Phospho-STAT5 (Tyr694) primary antibody (Cell signaling, 9359, IgG), cells were washed and resuspended in 20 µL 1x intracellular staining perm wash buffer containing Donkey anti-rabbit FITC-conjugated secondary antibody (BioLegend, 406403, IgG) for 30 minutes at 4 °C. Live/Dead fixable Near-IR dead cell stain kit (Invitrogen, L34976) was used to determine cell viability. All antibodies were added at the concentration recommended by the manufacturer. Thirty thousand events were acquired using FACS Canto flow cytometer (Becton Dickinson Immunocytometry Systems) with FACS Diva Software version 4.0.2. Data obtained were analysed using FlowJo software v7.6 (FlowJo, LLC). Data were summarised and analysed using Prism GraphPad 7 software. Statistical analysis was performed using one-way ANOVA.

Determining receptor expression

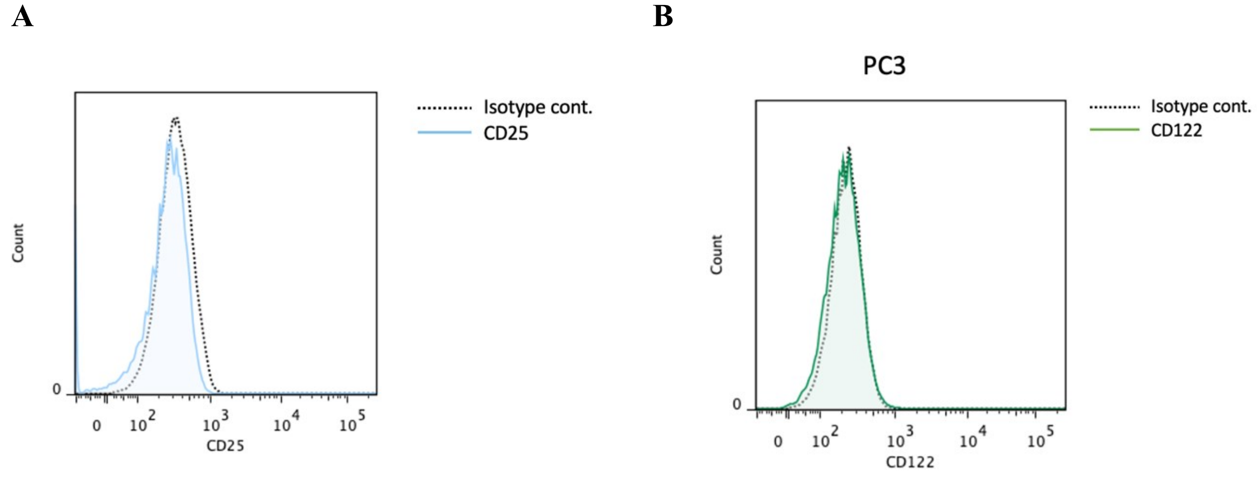

Figure 23. Representative histograms demonstrating cell surface expression of (**A**) CD25 (blue line) and (**B**) CD122 (green line) in PC3; three independent experiments were performed. Dotted line represents cells stained with isotype control antibody.

Determining phosphorylated STAT5

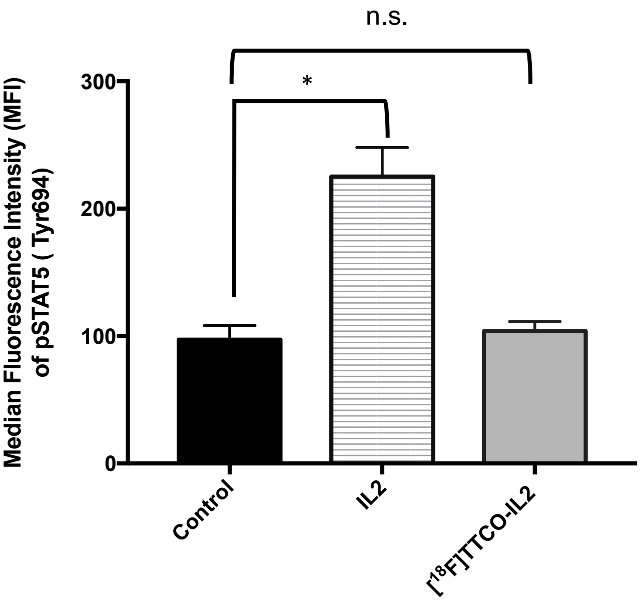

Figure 24. [^{18}F]TTCO-IL2 exerts no significant effect on STAT5 phosphorylation in NK-92 cells. Phosphorylation of STAT5 (Tyr694) in untreated NK-92 cells and NK-92 treated with recombinant IL2 (214 ng/mL) or [^{18}F]TTCO-IL2 (214 ng/mL) for 1 hour at 37 °C was measured with flow cytometry. Median fluorescence intensity (MFI) values from three independent experiments are presented. Data expressed as mean ± SEM. Statistical difference is analysed using one-way ANOVA, * (*$P \leq 0.05$).*

References

(1) Yang, J.; Karver, M. R.; Li, W.; Sahu, S.; Devaraj, N. K. Metal-Catalyzed One-Pot Synthesis of Tetrazines Directly from Aliphatic Nitriles and Hydrazine. *Angew. Chemie - Int. Ed.* **2012**, *51* (21), 5222–5225. https://doi.org/10.1002/anie.201201117.
